# HeLP: The Hebrew Lexicon project

**DOI:** 10.3758/s13428-024-02502-4

**Published:** 2024-09-09

**Authors:** Roni Stein, Ram Frost, Noam Siegelman

**Affiliations:** 1 Department of Psychology, The Hebrew University of Jerusalem, Mount Scopus Campus, 9190501 Jerusalem, Israel; 2 BCBL, Basque Center of Cognition, Brain and Language, San Sebastian, Spain

**Keywords:** Visual word recognition; Mega studies; Reading; Cross-linguistic differences

## Abstract

Lexicon projects (LPs) are large-scale data resources in different languages that present behavioral results from visual word recognition tasks. Analyses using LP data in multiple languages provide evidence regarding cross-linguistic differences as well as similarities in visual word recognition. Here we present the first LP in a Semitic language—the Hebrew Lexicon Project (HeLP). HeLP assembled lexical decision (LD) responses to 10,000 Hebrew words and nonwords, and naming responses to a subset of 5000 Hebrew words. We used the large-scale HeLP data to estimate the impact of general predictors (lexicality, frequency, word length, orthographic neighborhood density), and Hebrew-specific predictors (Semitic structure, presence of clitics, phonological entropy) of visual word recognition performance. Our results revealed the typical effects of lexicality and frequency obtained in many languages, but more complex impact of word length and neighborhood density. Considering Hebrew-specific characteristics, HeLP data revealed better recognition of words with a Semitic structure than words that do not conform to it, and a drop in performance for words comprising clitics. These effects varied, however, across LD and naming tasks. Lastly, a significant inhibitory effect of phonological ambiguity was found in both naming and LD. The implications of these findings for understanding reading in a Semitic language are discussed.

**Supplementary information:**

The online version contains supplementary material available at 10.3758/s13428-024-02502-4.

## Introduction

The ability to rapidly identify printed sequences of letters as individual words and automatically access their phonological and semantic representations has intrigued scientists for decades, and is still the focus of extensive research in cognitive science. Tasks that measure participants reaction times (RTs) and recognition accuracy of printed letter sequences have provided important insights regarding the computations underlying visual word recognition. Perhaps the most common experimental paradigm in such studies is the lexical decision (LD) task, where participants are presented with letter strings, one at a time, and are required to provide fast responses as to whether or not they represent existing words. Another common task is the naming task, in which participants are required to pronounce, as fast and as accurately as possible, a visually presented word. RT and accuracy data using the LD and naming tasks were taken to reveal the underlying computations in recognizing printed words presented in isolation, retrieving their phonological structure, and accessing their semantic representation. Across many studies, they demonstrated highly replicable effects which today are the landmarks of visual word recognition: As a non-exhaustive list, words are recognized faster than nonwords (i.e., the lexicality effect, e.g., Forster & Chambers, 1973; Monsell et al., 1989); frequent words are recognized faster than infrequent words (i.e., the word frequency effect, e.g., Broadbent, 1967; Brysbaert et al., 2017); shorter words are processed faster than longer words (i.e., the word length effect, e.g., Fredriksen & Kroll, 1976; Hudson & Bergman, 1985); and the number and frequency of orthographic neighbors affect decision times (i.e., the neighborhood density effect; e.g., Andrews, 1992; Grainger et al., 1989).

### Lexicon projects in different languages

In the first decades of visual word recognition studies, researchers typically employed experimental designs in which verbal stimuli were selected to represent factors of interest (e.g., imageability, concreteness, morphological complexity, homography, homophony), measuring performance for these stimuli. Generally, these studies used a relatively small set of stimuli, often not representative of the variety found across the full lexicon, thus limiting their external validity. One prominent example is the focus on monosyllabic words in English in visual word recognition studies, despite the fact that in many languages they represent a small portion of the words in the language (e.g., less than 15% of words, Ferrand et al., 2010).

An elegant solution to these limitations was the English Lexicon Project (ELP, Balota et al., 2007), which presented an open large-scale data resource that included over 40,000 words along with their respective behavioral data in both the LD and the naming tasks. The ELP made it possible, for the first time, to test a range of hypotheses regarding word recognition computations without the need to construct a targeted experiment with its inevitable limitations. Instead, researchers could generate hypotheses regarding the impact of any variable, and simply extract the behavioral data for all relevant stimuli from the database (a process sometimes referred to as “virtual experiments”, see, e.g., Kuperman, 2015). The ELP has been used extensively since its publication in order to explore the underlying computations of word recognition in English, and to date has been cited over 3000 times. It has been useful in validating and testing the impact of a range of psycholinguistic factors on reading, such as words’ semantic transparency (Kim et al., 2018), word length (e.g., New et al., 2006), imageability (e.g., Dymarska et al., 2023); orthographic–phonological regularities (e.g., Chee et al., 2020; Siegelman et al., 2020), and orthographic–semantic consistency (Siegelman et al., 2022).

Importantly, the ELP has also inspired the creation of parallel lexicon projects (LPs) in other languages, to provide a critical cross-linguistic perspective in reading research. These LPs include British English, to be distinguished from American English (Keuleers et al., 2012); Dutch (Keuleers et al., 2010); French (Ferrand et al., 2010); Spanish (Aguasvivas et al., 2018); Persian (Nemati et al., 2022); Malay (Yap et al., 2010); German (Schreuter & Schroeder, 2017); Portuguese (Soares et al., 2019); and Chinese (Tse et al., 2017). Together, the wide scale of cross-linguistic data offered by the different LPs have provided important insights into how basic word recognition processes vary across languages, and how the properties of a given writing system shape the cognitive computations involved in reading one language compared to another. From this perspective, the specific characteristics of a writing system can be taken as an “experimental manipulation” to examine their impact on reading performance (see Frost, 2012, for discussion).

But note that whereas all LPs have the same aim, methodologically there is substantial variability in how they were constructed. For example, while all available LPs provide LD data, not all include naming data. Further, although all LPs do provide a broader coverage of a language’s words than typical experiments, there is still substantial variability in the number of words they employed (e.g., from 1800 words and nonwords in Persian, Nemati et al., 2022; to 40,481 words and nonwords in English, Balota et al., 2007). LPs also differ in the number of participants providing responses to each word in the project (e.g., from 25 responses per word in the French LP, Ferrand et al., 2010, to 300 in the Spanish LP, Aguasvivas et al., 2018), and in other design characteristics such as the word–nonword ratio in the LD task. Table 1 summarizes the methodological properties of existing LPs, as well as the characteristics of the languages studied.

**Table 1:** A summary of large-scale Lexicon Project in different languages, and the properties of those languages

	Linguistic characteristics				Lexicon project details			
Lexicon Project	Topological family (branch)	Script (script type)	Morphological typology	Orthographic transparency	Number of words	LD/naming task	Average observations per word	Comments
**Chinese, Mandarin** (simplified script) (Tsang et al., 2018)	Sino-Tibetan (Sinitic)	Chinese (logographic)	Analytic	Opaque	12,578	LD	42	See also Sze et al., 2014
**Chinese, Cantonese** (traditional script) (Tse et al., 2017)	Sino-Tibetan (Sinitic)	Chinese (logographic)	Analytic	Opaque	25,286	LD	33	All stimuli were two-character compound words
**Dutch** (Keuleers et al., 2010)	Indo-European (West Germanic)	Latin (alphabetic)	Synthetic, fusional	Moderate	14,089	LD	39	Monosyllabic and disyllabic words
**English (American)** (Balota et al., 2007)	Indo-European (West Germanic)	Latin (alphabetic)	Moderately analytic	Opaque	40,481	LD & naming	LD- 34, Naming- 25	
**English (British)** (Keuleers et al., 2012)	Indo-European (West Germanic)	Latin (alphabetic)	Moderately analytic	Opaque	14,365	LD	39	Monosyllabic and disyllabic words
**French** (Ferrand et al., 2010)	Indo-European (Romance)	Latin (alphabetic)	Moderately analytic	Moderate	38,840	LD	25	
**German** (Schröter & Schroeder, 2017)	Indo-European (West Germanic)	Latin (alphabetic)	Synthetic, fusional	Moderate	1152	LD & naming	LD- 18, Naming- 20	Developmental project: Participants from 1st graders to adults
**Malay** (Yap et al., 2010)	Austronesian (Malayo-Polynesian)	Latin (alphabetic)	Synthetic, agglutinative	Transparent	9592	LD & naming	44	Participants got feedback if they were wrong
**Persian** (Nemati et al., 2022)	Indo-European (Western Iranian)	Arabic	Synthetic, fusional	Abjad	1800	LD		Words 3–8 letters long
**European Portuguese** (Soares et al., 2019)	Indo-European (Romance)	Latin (alphabetic)	Synthetic, fusional	Opaque	1920	LD & naming	55	
**Spanish** (Aguasvivas et al., 2018)	Indo-European (Romance)	Latin (alphabetic)	Synthetic, fusional	Transparent	44,853	LD	333	Word to nonwords ratio of 7:3

In spite of the substantial methodological variability, large-scale LP data have nonetheless largely replicated multiple well-established visual word recognition effects across languages, demonstrating substantial similarities in computations. For example, LPs have consistently revealed frequency and lexicality effects, as well as orthographic neighborhood density effects. Importantly, however, the multilingual comparisons that LPs inspired have also demonstrated significant differences in reading behavior across languages, providing illuminating insights into how the properties of a language and its writing system shape the computations involved in processing printed information. For example, LD data from the Persian LP showed no word length effect, in contrast to other LPs. This has led authors to assume that the specific properties of the Persian orthography—specifically, the under-specification of vowels in the printed language or the strong correlation between word length and orthographic neighborhood size—are the reason for the absence of the word length effect (Nemati et al., 2022). In stark contrast to Persian, the Malay LP revealed that the length of words in Malay is the strongest predictor of word recognition time. This again was tied to the structure of the writing system: the Malay language has a shallow orthography, transparent morphology, and a simple syllabic structure, which presumably lead Malay readers to rely primarily on serial conversion of letters to phonemes during word recognition. Indeed, further analysis of the Malay LP revealed great differences between Malay and English in the contribution of predictors such as frequency, orthographic neighbors, and word length to word recognition performance (Yap et al., 2010).

The cross-linguistic variations revealed by large-scale data highlight how the basic properties of a writing system impact word recognition behavior across languages, leading to a deep understanding of the universal principles involved in orthographic processing across the world’s languages. Since languages naturally differ in their scripts and how their writing systems represent sound and meaning, a good theory of reading should be able to explicate how this variation impacts the processing of printed words. When a given language diverges from others in terms of readers’ performance, important evidence is furnished regarding universal principles of reading (see Frost, 2012, for discussion). Such research enterprise, however, requires large-scale data from many diverse writing systems. The main goal of the present mega-study is to contribute to this important research effort by providing, for the first time, systematic data from a Semitic language—Hebrew—a language that has often been shown to produce contrastive results relative to European languages.

### The Hebrew language: An important test case

Hebrew is a Semitic language, as are Arabic, Amharic, and Maltese. Many words in Semitic languages are root-derived, so that their base is a root morpheme, usually consisting of three consonants, which conveys the core meaning of the word. Semitic words are constructed by intertwining root morphemes with word pattern morphemes—abstract phonological structures, consisting of vowels or of vowels and consonants, in which there are “open slots” for the root’s consonants to fit into. In general, word patterns provide vague morphosyntactic information. For example, in Hebrew, the root K.Š.R., which conveys the general notion of “tying”, and the word pattern /ti–o-et/, which is mostly used to denote feminine nouns, form the word /tikšoret/, meaning “communication”. Embedding the root K.Š.R. in the word pattern /-i-u-/ produces the word /kišur/, meaning “link”, etc. The root consonants can be dispersed within the word in many possible positions, and there is little a priori information regarding their location. Word patterns have a well-defined internal structure. Their onset comprises a restricted number of consonants (mainly /h/, /m/, /t/, /n/, /l/), and the order and identity of subsequent consonants and vowels is rigid. Because there are no a priori constraints regarding the location of root consonants in the word, the main clue regarding their identity is the well-defined phonological structure of the word pattern that allows the root consonants to stand out (Deutsch et al., 1998, 2021; Lador-Weizman & Deutsch, 2022). Overall, there are about 3000 roots in Hebrew, about 100 nominal word patterns, and seven verbal patterns.

Another major characteristic of the Hebrew writing system is its extreme phonological under-specification. Hebrew print consists of 22 letters, of which five have a finite letter form. The letters represent mostly consonantal information, and most vowel information is not conveyed in print (see Shimron, 2006; Ravid, 2011, for review). Two letters—one for both /o/ and /u/, and one for /i/—may convey the vowel information; however, in certain contexts, these letters also convey the consonants /v/ and /y/, respectively. This results in heavy phonological decoding demands, since a substantial part of the phonological information is missing. The missing vowels lead to an extensive homography, with many printed letter strings having multiple pronunciations and meanings (e.g., the printed word “ספר”, “SFR”, most commonly read as /sefer/, has seven possible pronunciations, each with a different meaning, depending on different vowel configurations). But since the structure of spoken words is highly constrained by the relatively small number of Semitic word patterns, readers can converge on a given word quite easily during text reading, since the printed form typically determines the appropriate word pattern with relatively high reliability, and once a word pattern has been recognized, the full vowel information is available to the reader, even if it is not specified by the printed form (Deutsch et al., 2021; Frost, 2006). Hence, Hebrew print provides a perfect example of optimization of information, where substantial morphological (and therefore semantic) information is provided along with sufficient phonological cues using minimal orthographic symbols (see Frost, 2012).^1^

From the perspective of orthographic depth (Frost et al., 1987; Katz & frost, 1992; Schmalz et al., 2015), Hebrew is considered to have a very deep orthography, given multiple features related to an underrepresentation of phonology in print. The first is the missing vowel information discussed above. In contrast to the depth of the English writing system, which results mainly from phonological inconsistency of vowel letters (e.g., EA is pronounced differently in DEAR, HEAD, and STEAK), in Hebrew, the vowel information is generally not inconsistent but missing. This poses a challenge in measuring and defining the phonological uncertainty of Hebrew words (Frost, 1994, 1995). A second source of phonological uncertainty in the Hebrew orthography is a feed-forward and feed-backward inconsistency of a few consonantal letters, where some can be mapped to different phonemes, and some phonemes can be represented by different letters. For example, the Hebrew letter “כ” can be pronounced as /x/ or /k/, ״ב״ can be pronounced as /v/ or /b/, and ‘פ’ can be pronounced as /f/, or /p/. Conversely, /t/ can be written with the letters "ת"or "ט", /s/ can be written as “ס” and “ש”, /k/ can be written as ‘כ’’ and “ק”, and /x/ can be written as “כ” and “ח”.

Lastly, another important feature of Hebrew is that morpho-syntactic information that is conveyed in European languages by function words (e.g., “the”, “from”, “to”, “and”) is conveyed in Hebrew as single letters which are attached to the word (i.e., clitics; e.g., “the” =  > ה, “from “ =  > מ, “to “ =  > ל, “and” =  > ו). For example, the four-word English sequence “and from the house” is printed in Hebrew as one word, where the three letters conveying and/from/the are attached to the base word “house” (“״ומהבית, printed as VMHBYT, read as /vemehabayit/, see Ravid, 2011). Clitics are abundant in Hebrew printed input, although their impact on visual word recognition is currently largely unknown. This is due to the tendency of previous studies in the word recognition literature (in Hebrew as in other languages) to focus on the processing of base words, rather than on more naturalistic stimuli which better reflect the distribution of printed words in a language.

### Visual word recognition in Hebrew: A series of divergent findings

Given the unique characteristics of the Hebrew writing system, it has been the focus of extensive research investigating how these impact reading. In fact, Hebrew has been used as a case study for investigating a variety of relevant domains, such as the predictors of eye movements during reading (e.g., Deutsch et al., 2003; Velan et al., 2013), the study of the developmental trajectory of literacy (e.g., Share, 1999; Share & Bar-on, 2018), the impact of different scripts on reading in a second language (e.g., Mor & Prior, 2020, 2021), and how the orthographic structure of Hebrew is reflected in various forms of dyslexia (e.g., Friedmann & Lukov, 2008; Friedmann & Rahamim, 2007). Given the focus on the current study, however, our review centers on how the properties of the Hebrew writing system impact visual word recognition processes. Generally, and in line with earlier studies in English and other languages described above, previous work has tended to focus on how specific features of the Hebrew orthography (e.g., homography, phonological ambiguity, morphological structure) lead to divergent patterns of visual word processing. Then, the impact (or lack thereof) of the studied feature was discussed within a broader framework, towards understanding the universal principles that drive word recognition processes across languages. Underlying this research agenda is the theoretical supposition that if language A (e.g., English) shows a given pattern of behavior, and language B (e.g., Hebrew) does not, this points to a higher-order principle that simultaneously explains both phenomena.

Within this research enterprise, substantial work has focused on Hebrew’s extreme phonological under-specification and homography. Some studies suggested that lexical decisions in Hebrew are made prior to phonological disambiguation (Bentin & Frost, 1987), and reflect the computation of a phonological impoverished code (see Frost, 1998, for discussion). Using the naming task, Frost et al. (1987) showed that frequency and semantic priming effects are stronger in Hebrew than in languages with shallower orthographies, resembling the effects revealed in LD. These results were taken to indicate that readers of languages with deep orthographies such as Hebrew rely more heavily on lexical information than readers of languages with shallow orthographies (e.g., Finnish, Spanish, German, Dutch), in order to compute phonology from print. This early finding is in line with the claim that readers of different writing systems rely on different informational cues in light of their writing system’s structure (e.g., Hirshorn & Harris, 2022; Lallier & Carreiras, 2018; Rau et al., 2015; Seidenberg, 2011; Seymour et al., 2003). However, all visual word recognition studies in Hebrew involved only a few dozen words that were selected as stimuli in each of the experiments. Moreover, most of these studies focused on nouns, typically disyllabic, without clitics or inflections—a partial set of stimuli which do not represent the variety of words in the Hebrew language.

In the same vein, extensive work has focused on how the morphological structure of Hebrew words affects visual word recognition (e.g., Deutsch et al., 1998; Feldman et al., 1995; Frost et al., 1997, 2000). Overall, these studies suggested that the root consonants are the core target of word recognition, and that lexical organization in Hebrew follows morphological principles. For example, Frost and colleagues (2005) showed that in contrast to English, French, or Spanish, full orthographic overlap between primes and targets in Hebrew results in very weak masked orthographic priming, interpreted as evidence that the lexical architecture of Hebrew probably does not align, store, or connect words by virtue of their full sequence of letters. Indeed, considering the overall body of research using masked priming in Semitic languages, reliable facilitation is consistently obtained whenever primes consist of the *root letters*, irrespective of what the other letters are (e.g., Frost et al., 1997, 2000; Velan et al., 2005; Perea et al., 2010, but see Perea et al., 2014, for significant form priming effects in Arabic).

Another important finding concerns letter position flexibility. In Indo-European languages, disrupting the order of the letters within a word has little impact on readers’ ability to recognize and read it correctly (e.g., Duñabeitia et al., 2007; Perea & Carreiras, 2006a, 2006b, 2008; Perea & Lupker, 2003, 2004; Schoonbaert & Grainger, 2004). In contrast, Hebrew readers reveal extreme letter position rigidity, and reading is significantly impaired when words involve transposed letters (Velan & Frost, 2007, 2009, 2011; and see Friedmann & Gvion, 2001, 2005, for letter position dyslexia of Hebrew readers). This cross-linguistic difference in letter position flexibility again reflects the morphological structure of Hebrew: since many Hebrew roots share a subset of letters but differ in their order (e.g., Z.M.R., “to sing”; R.M.Z., “to hint”; Z.R.M., “to flow”), letter position in Hebrew must be rigid rather than flexible in order to access the correct root (see Lerner et al., 2014, for computational evidence, and the recent PONG model, Snell, in press). However, to complicate things ever further, not all Hebrew words have a Semitic structure. Many words from various origins (e.g., Greek, Persian, English) have permeated Hebrew throughout history, and are not root-derived (and see a similar state of affairs for Maltese, e.g., Geary & Ussishkin, 2018). Indeed, these words have been shown to be processed differently from Semitic Hebrew words (Bitan et al., 2020; Haddad et al., 2018; Velan & Frost, 2011; Velan et al., 2013). However, the distributional properties of such non-Semitic Hebrew words are not yet known, and will be examined in this work.

### The current study: The Hebrew Lexicon Project

Since the Hebrew writing system represents a stark contrast to other alphabetic orthographies, and because word recognition experiments in Hebrew have revealed divergent findings with important cross-linguistic implications, contrasting Hebrew reading performance with other languages promises to provide important insights for reading research. Hence, a database of behavioral data on Hebrew words has far-reaching implications. Here we present an open data source on Hebrew visual word recognition, the Hebrew Lexicon Project (HeLP), which is the first to examine printed word recognition in a Semitic language on a large scale. The project reports data from two tasks: LD and naming. It assembles LD responses to 10,000 words and nonwords, with 5000 of the words also having additional associated naming data. Importantly, HeLP employs an ecologically valid set of Hebrew words sampled from a natural Hebrew printed corpus (see Methods), so that all types of words are included, including words with clitics, prefixes, and suffixes, Semitic and non-Semitic, inflected and derived—a variety that reflects the words Hebrew readers encounter in their daily lives.

In line with previous mega-studies and open-science studies more broadly, HeLP is meant to enable researchers to tackle a large number of questions regarding word recognition in Hebrew and its similarities and differences to other languages. The goal of this first paper, of course, is not to cover all such potential explorations. Rather, in the current paper, we demonstrate the utility of the HeLP data by addressing a series of foundational questions related, on the one hand, to the structure of the Hebrew writing system, and on the other to the predictors of word recognition in that language. In particular, as detailed below, our analyses examine the prevalence and impact of phonological uncertainty and homography in LD and naming tasks, the distributions of word lengths and neighborhood densities and their impact in a root-based orthography, and the distribution and behavioral consequences of Semitic and non-Semitic structure, as well as the number of clitics. Together, these analyses result in mapping, on a large scale, the behavioral impact of general predictors thought to reflect word recognition across languages (lexicality, frequency, word length, orthographic neighborhood density), but also predictors that are relevant specifically to reading in Hebrew (Semitic structure, presence of clitics, and phonological ambiguity).

The structure of the paper is as follows: First, we present results confirming the reliability of the HeLP data, using split-half estimates (at both the item and participant level), meant to ensure that the collected data are of sufficient quality for subsequent analyses. We then introduce descriptive statistics for the data, and present the basic effects revealed in the LD and the naming tasks pertaining to both general and Hebrew-specific predictors, as reviewed above. Finally, the implications of the results are discussed.

## Methods

### Lexical decision task

#### Stimuli

The project assembled LD data for 10,000 Hebrew words and 10,000 nonwords. Words were sampled from the Hebrew portion of the Subs2vec corpus (van Paridon & Thompson, 2021) which has 170 million tokens. First, a list of the 50,000 most frequent Hebrew words was extracted from the corpus, and all proper names and misspelled words were manually removed from that list. We then randomly sampled 2500 words from the 5000 most frequent words in the list, and 7500 words from the remainder of the frequency range of the filtered list, which together made the 10,000 targets for the LD task.

Nonwords were generated by shuffling letters of all words from the 50,000-word filtered list. Words with four or fewer letters had all their letters reshuffled. Words with five or more letters had their beginning, middle, or end shuffled randomly (first, middle, or last letters of the word). The number of letters shuffled ranged from 3 to *n* − 1. Following this procedure, 10,000 nonwords were selected randomly and then inspected manually to ensure they truly had no meaning in Hebrew. The shuffling of different numbers of letters at different locations within words avoided manual decisions that may introduce bias, and was meant to provide a variety of items which could be mined to study the determinants of ease or difficulty in rejecting letter strings as potential Hebrew words. As such, our “shuffling” approach resulted, for example, in items that have a clearer expected pronunciation (e.g., ביגלרת, טגל), along with others that are more phonologically ambiguous (e.g., רמלש); in nonwords with a pseudo-morphological Semitic structure (e.g., האחבטה, להגהר); and in nonwords that vary in bigram frequency. In this first paper we do not analyze predictors of responses to nonwords, but the nonword data are made fully available with the rest of the dataset for future research.

**Fig. 1:**
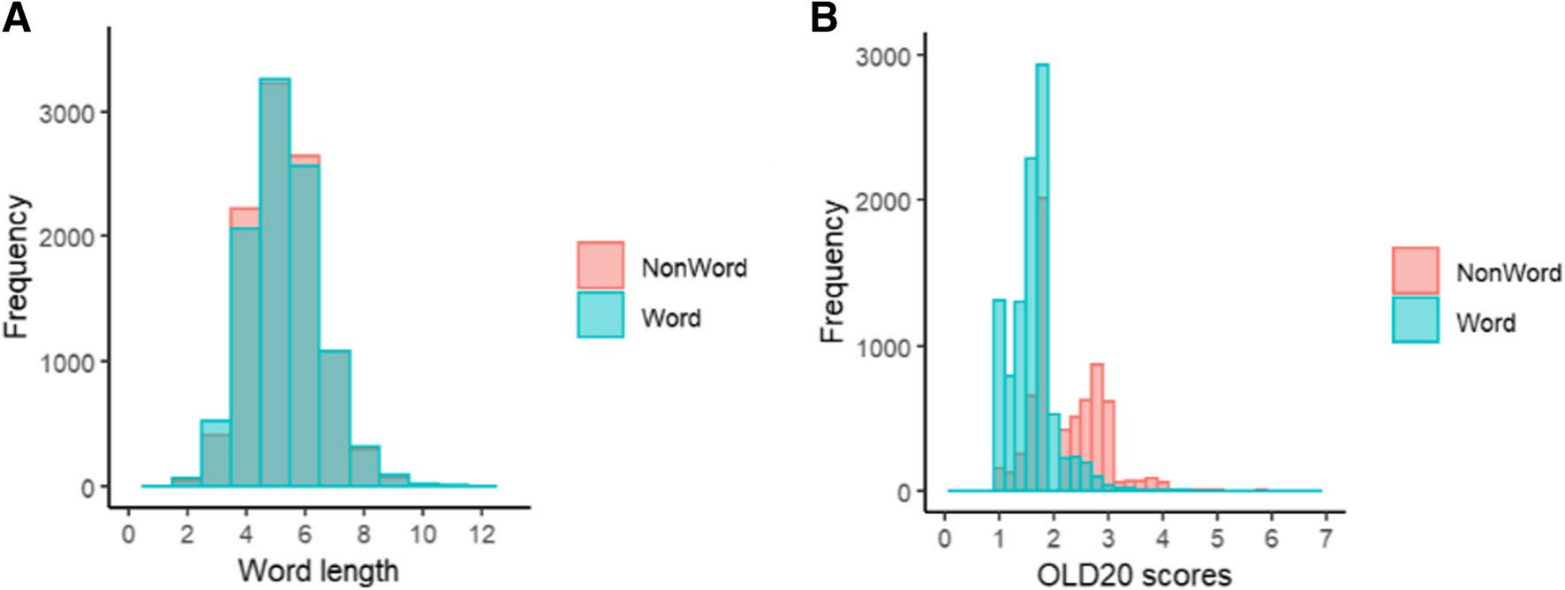
Properties of words and nonwords in the LD task*.* **A** Distribution of length (in letters). **B** Distribution of OLD20

Twenty sublists, each comprising 500 words and 500 nonwords (1000 targets overall), were created to serve as stimuli for each experimental session in the LD task. To ensure that each sublist included words from the full frequency range, the first word was assigned to the first sublist, the second word to the second sublist, etc., and the 21st word was assigned again to the first sublist and so on. Results from the 20 sublists were analyzed to examine the general effects such as lexicality (see Supplementary Materials S1). Since Hebrew-specific predictors required manual coding, only words from the 10 sublists that were also employed in the naming task were used in analyses considering these predictors (see details on naming task stimuli, below). Nonwords were randomly assigned to the sublists, and Welch *t*-tests ensured that the words and the nonwords in every sublist did not differ significantly in terms of length (*t*(19,970) =  − 0.4, *p* = 0.68; see Fig. 1A). In contrast, words and nonwords did differ in their mean orthographic Levenshtein distance 20 (OLD20), a measure of orthographic neighborhood density defined as the mean Levenshtein distance of the 20 closest orthographic neighbors of an item (Yarkoni et al., 2008), with a mean OLD20 of 1.65 for words and 2.17 for nonwords (*t*(18,909) =  − 74.5, *p* < 0.001, see Fig. 1B). This is expected because words share more orthographic resemblance to other words, whereas nonwords (resulting from shuffling letters) have less resemblance to existing words.

#### Participants

A total of 273 participants completed the experimental sessions. Data for nine participants were excluded from the analysis due to technical difficulties and poor performance (mean accuracy lower than 75%, or more than 12% of responses under 300 ms). Overall, the results for 264 participants (201 female) had valid LD data. Due to manual coding of stimuli from only a partial set of overlapping lists that were employed in both the LD and naming tasks, our central models which include Hebrew-specific predictors were conducted on data from 178 participants. Participants were students at the Hebrew University of Jerusalem and were recruited using the Psychology Department’s participant recruitment platform. The average age of the participants was 24.14 years (*SD* = 3.53 years). All participants declared that they had no attention or reading disabilities, that their first language was Hebrew, and that they had normal or corrected-to-normal vision. Participants received credit or payment for their participation after each experimental session. Each participant could take part in as many experimental sessions as they wished, up to 20 (see below), but they were required to take at least a 15-min break between sessions, and could not take part in more than two sessions a day.

#### Procedure

In the LD task, all experimental sessions were performed online from home (using laptop or desktop computers only, i.e., not via smartphones or tablets). The LD task was built using PsychoPy (Peirce et al., 2019), version 2021.2.3, and hosted on Pavlovia. After signing a consent form and confirming eligibility criteria, participants were instructed that in each trial they would be presented with a letter string on the computer screen, to which they had to respond as rapidly and as accurately as possible as to whether it formed an existing Hebrew word (pressing the “L” key) or not (pressing the “S” key). The session began with 10 practice trials consisting of five words and five nonwords, followed by the experimental stimuli. Each stimulus remained on the screen until a response was recorded, with a timeout of two seconds, and a blank screen for 500 ms between stimuli. Targets were presented in the middle of the screen, in white font on a dark gray background, with their size set to 10% of a participant’s screen (the absolute dimensions varied given the online nature of the task). There were three breaks during the session, after 250, 500, and 750 trials. Each experimental session typically lasted between 20 and 30 min.

### Naming task

#### Stimuli

From the 20 sublists (i.e., 10,000 words) that were used in the LD task, we sampled 10 sublists, with a total of 5000 words to serve as stimuli in the naming task, maintaining the same frequency distribution as in the full set of 10,000 words. The 5000 words were redivided into six sublists for the naming experiment, four of them with 800 words and two with 900 words. Each of the six naming sublists included words from the full frequency range as described above (25% from the 5000 most frequent words, and 75% from the entire range of frequencies following the 5000 most frequent words).

#### Participants

A total of additional 151 participants (101 female) completed the naming experimental sessions, using the same recruitment procedure. The mean age of participants was 24.6 years (*SD* = 4.4 years). As in the LD task, participants could take part in as many experimental sessions as they wished (up to six, the number of sublists).

#### Procedure

In contrast to the web-based LD task, all experimental sessions in the naming task were performed in the laboratory. The naming task was built using the NeuroBehavioral Systems software, Presentation, version 23.010.27.21. Participants sat in front of a computer screen in a quiet experiment room, wearing a headset. They were told that Hebrew words would appear on the screen, one at a time, and they should read every word aloud as fast and as accurately as they could. Before each word, a fixation cross appeared in the middle of the screen for 800 ms. The word disappeared from the screen once a participant initiated a voice key with a spoken response or after a timeout of 1.5 s. The experiment started with 10 practice trials, to ensure that the voice key operated correctly (experimenters adjusted the threshold if needed). There were two breaks during the experiment, after 250 and 500 words in the 800-word sessions, and after 300 and 600 words in the 900-word sessions. Words were presented in the middle of the screen, in 55-pt. white font on a dark gray background, taking about 10% of the vertical dimension of the screen. RTs were measured from the appearance of a word on the screen to the activation of the voice key. Similar to the LD task, participants who wished to participate in multiple sessions had to take a break of at least 15 min between sessions, and could not participate in more than two sessions in one day. Each experimental session typically lasted around 30 min.

Responses in the naming task were recorded and were later coded by a team of five trained research assistants. Each response was coded both for its accuracy (correct/incorrect; and in rare cases, “unclear”—see below) and for the validity of the RT data (i.e., whether the first recorded auditory signal, which triggered the voice key, should be used in RT analysis). A response could be coded as correct/incorrect while still not having valid associated RT: coding as “invalid RT” was automatically assumed in cases where the responses were faster than 200 ms, as well as in additional cases where the coder noticed another (invalid) response that triggered the voice key (e.g., in cases where there was an initial sound such as a cough or murmur before the participant read the target). In such cases, we used the correctness data but not the RT data in the analyses below. A total of 94.5% of responses were coded as having a valid RT associated with them, whereas 99.4% of responses had a correct/incorrect coding (i.e., only 0.6% of responses had “unclear” as the coding for correctness; in analyses below, we treat these as NA in all models).

To ensure the reliability of the coding of naming responses, we further randomly sampled six sessions from six participants, with 5000 naming trials in total (four participants had 800 trials; two participants had 900 trials). The re-coding of these sessions was done by another (blinded) research assistant, who used the same coding scheme as the original coders. We found an inter-rater reliability estimate of Cohen’s $\kappa =0.62$, a value representing “substantial” agreement between raters (Landis & Koch, 1977), with estimates in the data from the six randomly sampled participants separately ranging from $\kappa =0.52$ to $\kappa =0.85$. These estimates suggest that the reliability of the naming response coding was substantial overall and at a minimum moderate in individual participants.

### Predictors of visual word recognition performance: Word-level variables

**Fig. 2:**
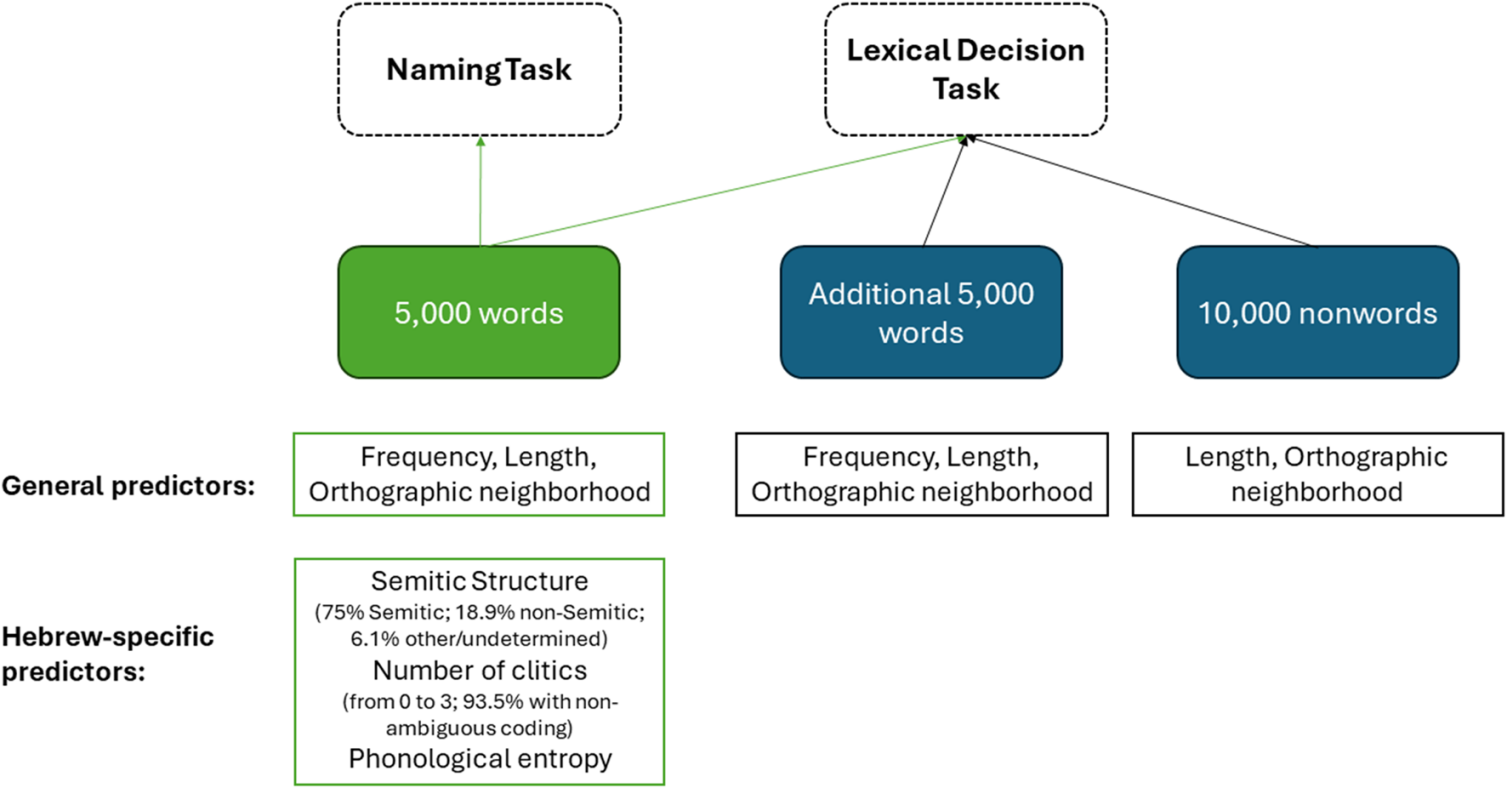
Information about included HeLP stimuli and their available lexical properties

Figure 2 summarizes the different psycholinguistic predictors made available for the different sets of stimuli with the current release of the HeLP data. In what follows, we provide more information about these predictors.

#### General visual word recognition predictors

The models below use as predictors the following general variables, which are known to impact word recognition across many languages: (a) *lexicality*, whether a target is a word or a nonword (in the LD task); (b) *word frequency* (string frequency of the surface form, log-transformed), based on the Subs2vec corpus (van Paridon & Thompson, 2021); (c) *word length* (in number of letters); and (d) *OLD20*, computed using the filtered list of 50,000 words from the Subs2vec corpus, examining for each item the number of substitution, insertion, or deletion operations required to turn that item into any of the other 50,000 words in the list. Then, OLD20 was defined as the mean number of alterations in the 20 words that required the minimal number of alterations (Yarkoni et al., 2008).

#### Hebrew-specific predictors

In addition to these general predictors, our models consider predictors potentially relevant specifically to word recognition in Hebrew (and other Semitic languages), given previous research and considering the properties of the writing system. As detailed below, obtaining these measures required excessive manual coding of responses. Hence, we focused on the 5000 words that were used as stimuli in both the naming and the LD tasks (rather than on the full set of 10,000 words in the LD task).

**Fig. 3:**
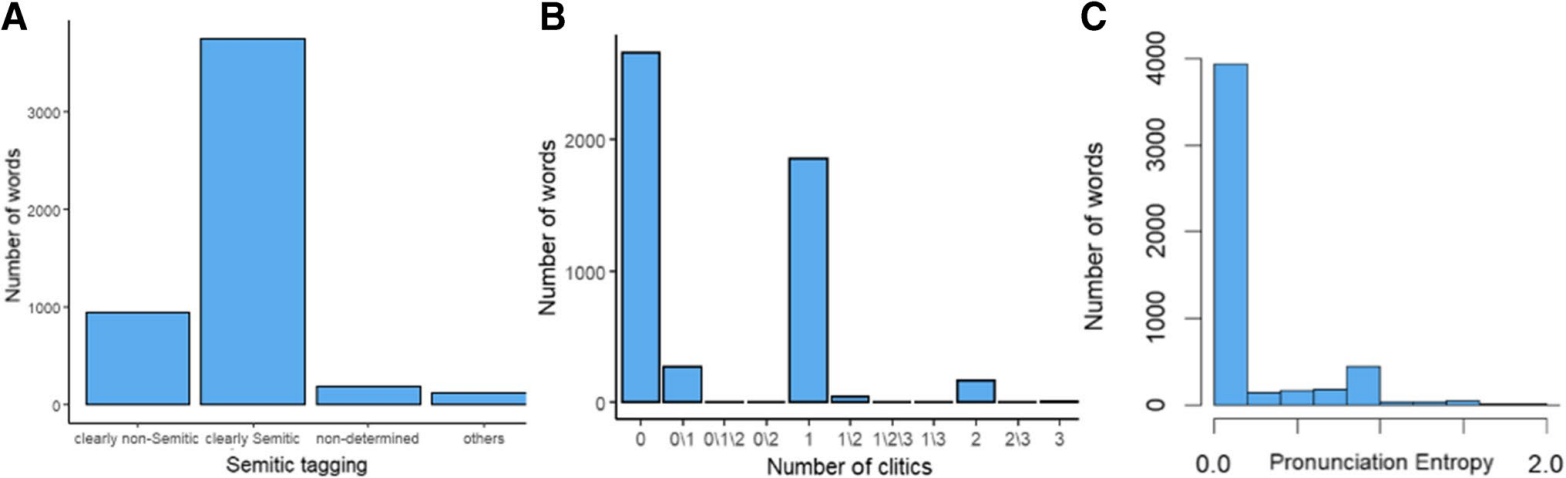
Distributions of the Hebrew-specific predictors.** A** Number of words tagged as 0-clearly non-Semitic, 1-clearly Semitic, 2-undetermined, 3-other. **B** Number of words with each number of clitics. **C** Pronunciation entropy scores

**Semitic structure.** As reviewed in the Introduction, an important feature of Hebrew is that while many words have a Semitic structure and comprise root and word pattern combinations, there are also many words without Semitic structure (see Velan & Frost, 2011), which have been assimilated into Hebrew throughout history. The Semitic tagging of words was manually performed such that each word was tagged into one of four categories: “clearly Semitic”, “clearly non-Semitic”, “undetermined”, or “other”. Words were classified as “clearly Semitic” if they had an unequivocal root which was productive and used in different phonological patterns, creating a variety of words with distinct meanings. For example, the word "כתבתי" (KTBTI, meaning I wrote) is constructed from the root letters “כ,ת,ב” (K.T.B.), and appears in many Hebrew patterns to create distinct words (e.g., "מכתב", MKTB, /mixtav/—a letter; "מכתיב", MKTIB, /maxtiv/—dictates; "כתב", KTB, /katav/—he wrote; see, e.g., Frost et al., 1997). Words were tagged as clearly non-Semitic if they could not be decomposed into a productive root and a word pattern (e.g., the word "לימון", LIMWN, /limon/, meaning a lemon, which was assimilated into Hebrew and is not derived from a productive root or pattern). The “undetermined” category was used for words that could be read or analyzed in more than one way (e.g., "אטום", ATWM, can be read as /atom/, meaning an atom, a non-Semitic word, or as /atum/ meaning “sealed”, derived from the root A.T.M.), or words that could not be unequivocally classified as having a Semitic structure. In the “other” category there were prepositions, adverbs, and pronouns, which do not follow either a Semitic or non-Semitic structure. Our tagging revealed that 75% of the 5000 words were clearly Semitic, 18.9% were defined as clearly non-Semitic, and 6.1% were undetermined/other (Fig. 3A). Only words with a clear Semitic or non-Semitic structure were included in the models below.

Coding of Semitic structure was performed by members of the same team of trained research assistants mentioned above. We further later randomly sampled a subset of 450 words, and asked another research assistant to code words using the same scheme, to estimate inter-rater reliability. Cohen’s $\kappa$ was estimated at 0.62, again reflecting “substantial” inter-rater agreement (Landis & Koch, 1977).

**Number of clitics**. As noted above, another important feature of Hebrew printed words is that they often involve clitic letters that carry functional information, which are attached to the word beginning, forming a single orthographic sequence. To capture this information, each word was manually tagged for its number of clitic letters (from 0 to 3). For the majority of words, the number of clitics was unambiguous (93.66% words had 0, 1, 2, or 3 clitics). However, since Hebrew print is phonologically ambiguous (i.e., about 25% of the words in an un-pointed written text are heterophonic homographs, see Shimron & Sivan, 1994), the number of clitics was sometimes not unequivocal (e.g., words with the initial letter H could often be interpreted as having the clitic “the”, but the same letter could also be an initial root/pattern letter; for instance, the word "הדלק", HDLK, can be read as \hadelek\, where the \ha-\ is the definiteness-marking form followed by the noun \delek\, “fuel”, as well as \hadlek\, where the initial H is part of the imperative form of the root D.L.K., “to light up”). The coding of these words is noted as X/Y (e.g., 0/1, meaning a word with either zero or one clitic, depending on its reading). Only words with a clear number of clitics were included in the models below. After removing words due to unclear Semitic and clitic tagging, data from 4441 words were analyzed. The distribution of clitics is presented in Fig. 3B. This coding was again performed by members of the research assistant team, and inter-reliability was estimated using the same sample of 450 words. Cohen’s $\kappa$ was estimated at 0.85, reflecting “near perfect” agreement (Landis & Koch, 1977).

**Phonological entropy.** As noted in the Introduction, another crucial feature of Hebrew is its extensive homography and ambiguity in terms of the orthographic–phonological mapping. That is, whereas some Hebrew words have one possible meaningful pronunciation, others can be read in multiple ways, and indeed, a substantial portion of words in the naming task were read in more than one way across participants. To capture this ambiguity, we calculated each word’s pronunciation entropy, based on the distribution of unique pronunciations across participants in the word-naming data (entropy is defined as $-\sum_{i=1}^{N}p(i)*{\text{log}}_{2}p(i)$, where $N$ is the number of different pronunciations of a word across participants, and $p(i)$ is the proportion of responses of a given pronunciation; see also De Simone et al., 2021). The more unique pronunciations a written word had in the naming data, and the more uniform the distribution was, the more pronunciation entropy increased. A total of 76.9% of the words in the naming task were pronounced in only one way and therefore their pronunciation entropy was zero (i.e., at least in the current behavioral data, these words were taken to be non-homographic). The remaining words had entropy values up to 1.97. The distribution of phonological entropy is shown in Fig. 3C.

### Data and code availability

The full HeLP data are available via the Open Science Framework (OSF) website for secondary data analyses. Two main data files include the LD and the naming data (“raw_LD_data.xlsx” and “raw_naming_data.xlsx”, respectively). These files include the trial-level behavioral data (accuracy and reaction time), as well as the values of general predictors (frequency, word length, OLD20). For the 5000 words used in the naming task, the files also include the values of the manually coded Hebrew-specific predictors (Semitic tagging, clitics, and pronunciation entropy). For items that were pronounced in more than one way across participants, we also make available the phonological transcription of the pronunciation at each trial. A third file (“Hebrew_words_data.xlsx”) lists the 10,000 words of HeLP along with their characteristics and tagging. The repository also includes the R codes used for running the analyses reported below. Please see: https://osf.io/nxq8g/.

## Results

### Data cleaning

#### Lexical decision task

**Fig. 4:**
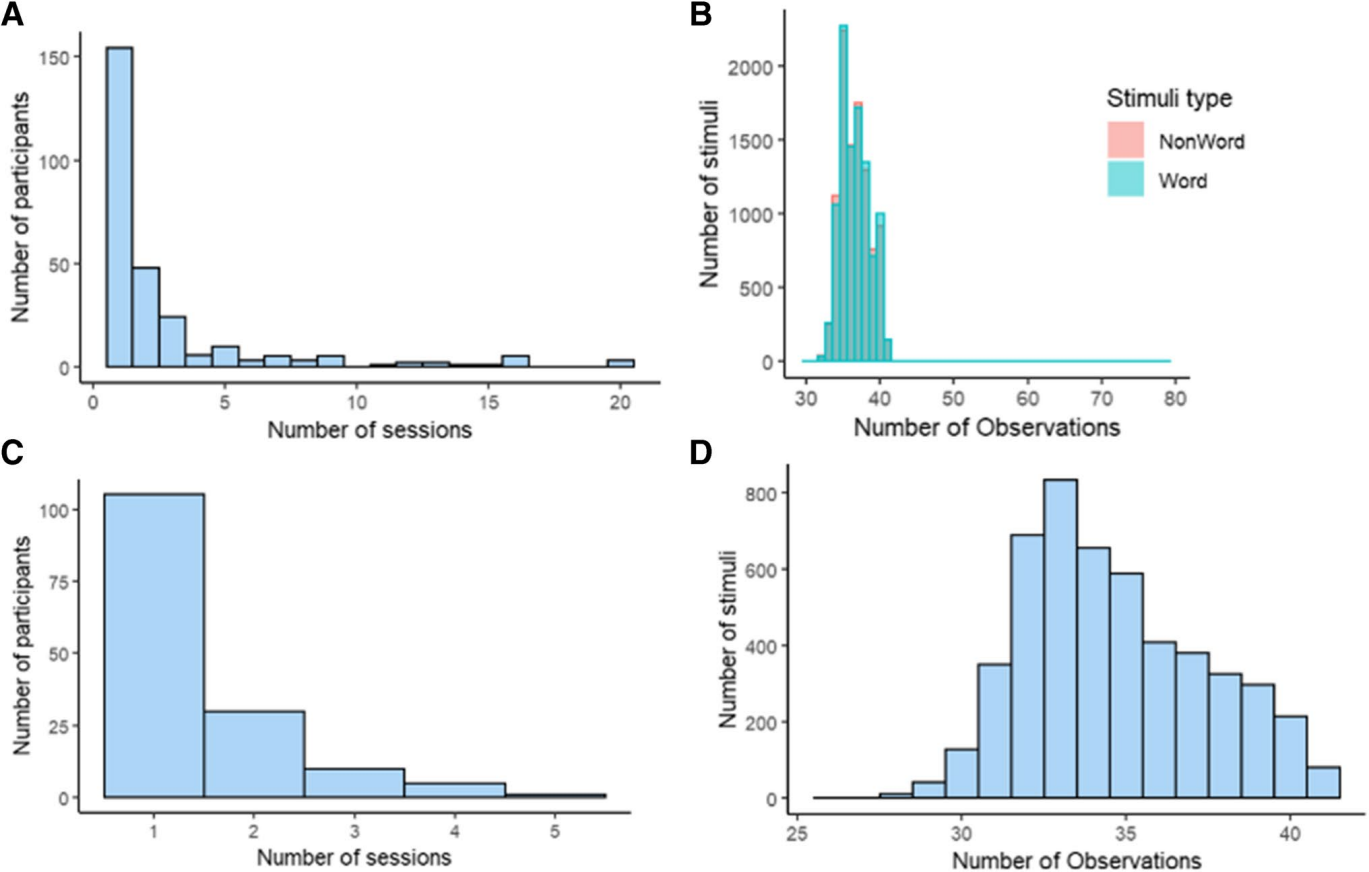
**A** Distribution of the number of sessions per participant in the LD task. **B** Distribution of the number of responses per word and per nonword in the LD task. **C** Distribution of the number of sessions per participant in the naming task. **D** Distribution of the number of responses per word in the naming task

Overall, there were 748 experimental sessions across participants. As noted above, participants could contribute data in more than one session: Participants took part, on average, in 2.7 experimental sessions, with 154 participants taking part in one session and, on the other extreme, three participants who completed all 20 sessions (see Fig. 4A for the distribution of the number of sessions by participant). Thirty-two sessions were discarded from the analysis, as follows: because of poor performance, with less than 75% accuracy (17); because the data included many trials with unrealistically fast RTs, defined as 12% of trials being faster than 300 ms (14); or due to technical difficulties (1). From the remaining sessions, all trials with RTs faster than 300 ms were also excluded (~ 2% of the trials). Therefore, the results of 716 experimental sessions were included in the cleaned dataset, with data from 710,507 LD trials. Across these sessions, the average number of participants that provided data for each stimulus was 35, while the minimal number of observations per stimulus was 29, and the maximum was 79,^2^ with S*D* of 2.5 (see Fig. 4B).

#### Naming task

A total of 220 experimental sessions were carried out. Participants took part on average in 1.4 experimental sessions (see Fig. 4C). RTs faster than 300 ms were removed from the analysis (~ 5% of the trials), as well as trials where there were no responses to stimuli (mostly due to technical difficulties, also ~ 1% of the trials). All participants had accuracy higher than 75%, and therefore, in contrast to the LD data, no full participants were discarded. This resulted in a total of 173,099 naming trials that were analyzed. The average number of participants that were exposed to each stimulus in the “clean” data was 35, while the minimum was 26 participants and the maximum was 41, with *SD* of 2.77 (see Fig. 4D).

### Descriptive statistics

**Fig. 5:**
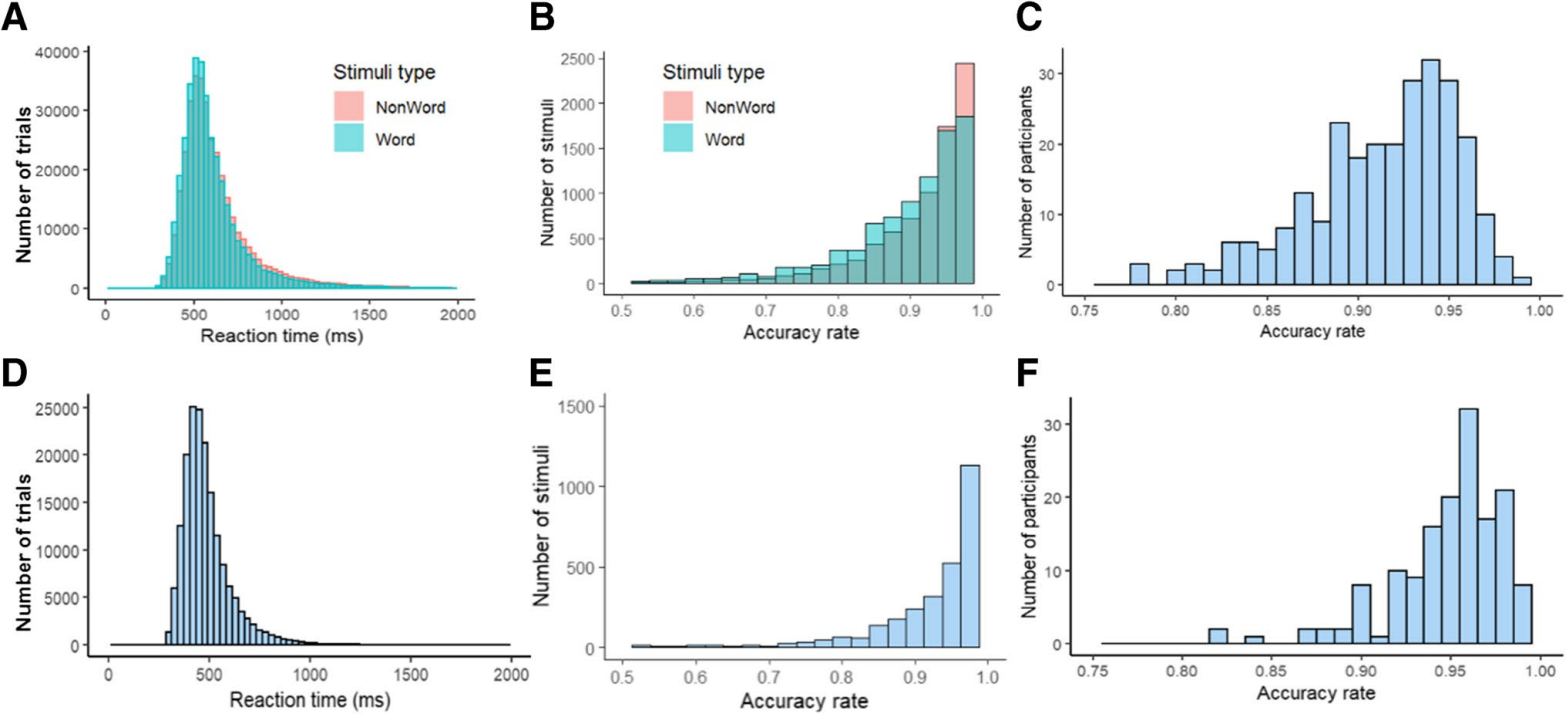
**A** Distribution of RT for words and nonwords in the LD task (across trials). **B** Distribution of accuracy rate for words and nonwords in the LD task. **C** Accuracy rates by participant in the LD task. **D** Distribution of RT for words in the naming task (across trials). **E** Distribution of accuracy rate for words in the naming task. **F** Accuracy rates by participant in the naming task

In the LD task (see Fig. 5A–C), mean accuracy across participants was 91.3% (range: 77.7–98.8%, *SD* = 4%). Mean accuracy was 92.4% for nonwords and 89.4% for words. The mean RT to words was 606 ms, and the mean RT to nonwords was 631 ms. In the naming task, mean accuracy was 94.86% (range: 81.85–99.28%, *SD* = 3%), with mean latency of 489 ms (see Fig. 5D–F).

### Reliability estimates

We examined the internal consistency of the data using a split-half reliability estimate with Spearman–Brown correction at both the subject and item levels. The consistency of each participant’s responses was examined by splitting trials to even and odd data points for each participant. Mean log-transformed RT and accuracy were calculated per participant for the two halves of the data. The split-half correlation between the means was calculated and corrected using the Spearman–Brown correction formula. In the LD task, participant-level reliability was 0.97 for accuracy and 0.99 for log-transformed RTs. Reliability at the stimulus (i.e., word/nonword) level was assessed by splitting *participants* into evens and odds, correlating the mean log-transformed RT and accuracy of each stimulus across the two groups of participants. The LD item-level split-half correlation was 0.79 for accuracy and 0.66 for log-transformed RT. For the naming task, consistency at the participant level was 0.96 for accuracy and 0.99 for log-transformed RT. Reliability at the stimulus (i.e., word) level was 0.85 for accuracy and 0.66 for log-transformed RT. Together, these reliability estimates suggest that our LD and naming data are highly stable when measuring individual differences in log-transformed RT and accuracy (i.e., very high split-half reliability at the subject level) and provide moderately to highly reliable estimates at the item level.

### Basic effects: Lexicality, word length, and neighborhood density

First, we used the full LD data—from both 10,000-word and 10,000-nonword targets—to estimate the impact of three basic predictors that apply to words and nonwords alike: lexicality (word vs. nonword), word length, and neighborhood density. This analysis was meant to serve as an initial validation of the HeLP data, and to examine basic visual word recognition predictors in Hebrew. We employed a linear mixed-effects model to predict log-transformed RT and a logistic mixed-effects model to predict accuracy, which included the three variables as fixed effects as well as all their two-way and three-way interactions, along with a fixed effect for session number (a control variable). Here and below, analyses of RTs were conducted only on trials with correct responses. In all models here and below, we used the *buildmer* R package (Voeten, 2019) to establish the maximal random-effects structure justified by the design that converged. In linear mixed-effects models, degrees of freedom and *p*-values were calculated using the *lmerTest* package in R via a Satterthwaite approximation (Kuznetsova et al., 2017).

The results of these models showed a significant effect of lexicality in both RT (i.e., faster responses to words than nonwords) and accuracy (i.e., more errors for words than for nonwords). Across words and nonwords together, length effects were also significant, indicating overall faster responses, but with more errors, in shorter stimuli. Importantly, however, the effects of length were modulated by lexicality (see Supplementary Materials S1, and subsequent analyses of word data, below). A significant effect of OLD20 was observed, suggesting faster and more accurate responses to stimuli with fewer orthographic neighbors, though here as well there were strong interactions with length and lexicality. The structure and results of the mixed-effects models are detailed in Supplementary Materials S1, which also includes a discussion of the interactions among the three variables.

### What predicts word recognition RT and accuracy in Hebrew?

The preliminary models above center on general effects in the LD task as preliminary validation of the data. In the remainder of the paper, we center on predictors of Hebrew word recognition, focusing on both general predictors (i.e., frequency, length, neighborhood density) and Hebrew-specific predictors (Semitic structure, clitics, extent of homography). For this purpose, all models in this section included data from 4441 Hebrew words—the number of stimuli used in both the LD and the naming tasks that had valid coding of all Hebrew-specific predictors (see Methods). We again used linear mixed-effects models for log-transformed RTs, and logistic models for accuracy. As predictors, all models in this section include word length, log frequency, OLD20, semitic tagging, number of clitics, pronunciation entropy, and session number^3^ as fixed effects. Interactions between word log frequency and word length, OLD20 and word length, and OLD20 and log frequency were also estimated. All continuous parameters were scaled, the Semitic/non-Semitic contrast was effect-coded and adjusted so that the mean of this variable was set to 0 (i.e., centered), and the number of clitics was entered into the model as a factor, using a dummy coding scheme (0 clitics set as the baseline). The random-effects structure of the models was again determined using the *buildmer* package (the maximal random-effects structure that converged is specified in each of the models below).

Fixed-effect estimates for our four central models (i.e., RT and accuracy, for LD and naming) are summarized in Table 2. Table 3 further shows the pairwise correlations between word-level numeric predictors in these models, to gauge collinearity in our models. In particular, note the strong collinearity between word length and OLD20 scores (*r* = 0.79). To examine how this collinearity impacts the results of our models, further attempts were made to isolate the word length and OLD20 effects by running the same models without the length predictor and the same models without the OLD20 predictor, to ensure that the findings below regarding these two predictors were not simply an outcome of this high collinearity. This process is detailed in Supplementary Materials S2, showing that in most cases the effects (or lack thereof) of length and OLD20 reported below are replicated qualitatively when these two measures are considered separately. However, when reviewing the models below, we highlight cases where this was not the case. There was also a weaker collinearity between log frequency and OLD20 (*r* =  − 0.44). Other correlations between predictors were not higher than |0.34|.

**Table 2:** Predictors of performance in LD and naming tasks (based on data from 4441 Hebrew words). *p < .05; **p < .01; ***p < .001

	Lexical decision: RT			Lexical decision: accuracy			Naming: RT			**Naming: accuracy**		
	Coefficient	*t* value	*p* value	Coefficient	*Z* value	*p* value	Coefficient	*t* value	*p* value	**Coefficient**	***Z* value**	***p* value**
**Log frequency**	− 0.035	− 23.847	< 0.001 ***	0.532	21.720	< 0.001 ***	− 0.014	− 11.896	< 0.001 ***	0.541	15.670	< 0.001 ***
**Word length**	− 0.012	− 6.061	< 0.001 ***	0.596	20.308	< 0.001 ***	− 0.004	− 2.303	0.022 *	− 0.126	− 2.748	0.006 **
**OLD20**	0.012	6.355	< 0.001 ***	− 0.108	− 3.510	< 0.001 ***	0.002	1.077	0.282	0.361	7.260	< 0.001 ***
**Semitic**	− 0.012	− 4.756	< 0.001 ***	0.235	5.770	< 0.001 ***	− 0.005	− 2.167	0.031 *	0.040	0.626	0.531
**Clitic–1**	0.010	4.745	< 0.001 ***	− 0.114	− 3.595	< 0.001 ***	− 0.021	− 11.949	< 0.001 ***	− 0.262	− 4.749	< 0.001 ***
**Clitic–2**	0.018	3.382	< 0.001 ***	− 0.035	− 0.420	0.675	− 0.023	− 5.050	< 0.001 ***	− 0.394	− 2.874	0.004 **
**Clitic–3**	0.008	0.289	0.772	− 0.521	− 1.264	0.206	0.055	2.238	0.025 *	− 1.985	− 3.292	< 0.001 ***
**Pronunciation entropy**	0.004	4.167	< 0.001 ***	− 0.033	− 2.314	0.021 *	0.006	6.085	< 0.001 ***	− 0.092	− 3.710	< 0.001 ***
**Word length × OLD20**	0.003	5.319	< 0.001 ***	− 0.070	− 7.659	< 0.001 ***	0.002	3.112	0.002 **	− 0.099	− 6.320	< 0.001 ***
**Log frequency × word length**	0.007	4.645	< 0.001 ***	− 0.156	− 6.328	< 0.001 ***	0.002	1.421	0.156	− 0.134	− 3.019	0.002 **
**Log frequency × OLD20**	− 0.006	− 3.331	< 0.001 ***	0.076	2.752	< 0.001 ***	− 0.001	− 0.783	0.434	0.054	1.083	0.279
**Session number**	− 0.029	− 27.091	< 0.001 ***	− 0.170	− 11.522	< 0.001 ***	− 0.006	− 7.724	< 0.001 ***	0.146	7.118	< 0.001 ***

**Table 3:** Pairwise Pearson correlations between numeric predictors across the 4441 words included in models reported in Table 2

	(1)	(2)	(3)	(4)
(1) OLD20		0.44	0.79	− 0.16
(2) Log frequency			− 0.34	− 0.02
(3) Word length				− 0.16
(4) Pronunciation entropy				

#### Evidence from lexical decision

**RT model.** The maximal mixed-effects model that converged predicting log-transformed RT included by-subject random slopes for word length, frequency, pronunciation entropy, semitic tagging, and OLD20, as well as by-subject and by-item random intercepts.

**Fig. 6:**
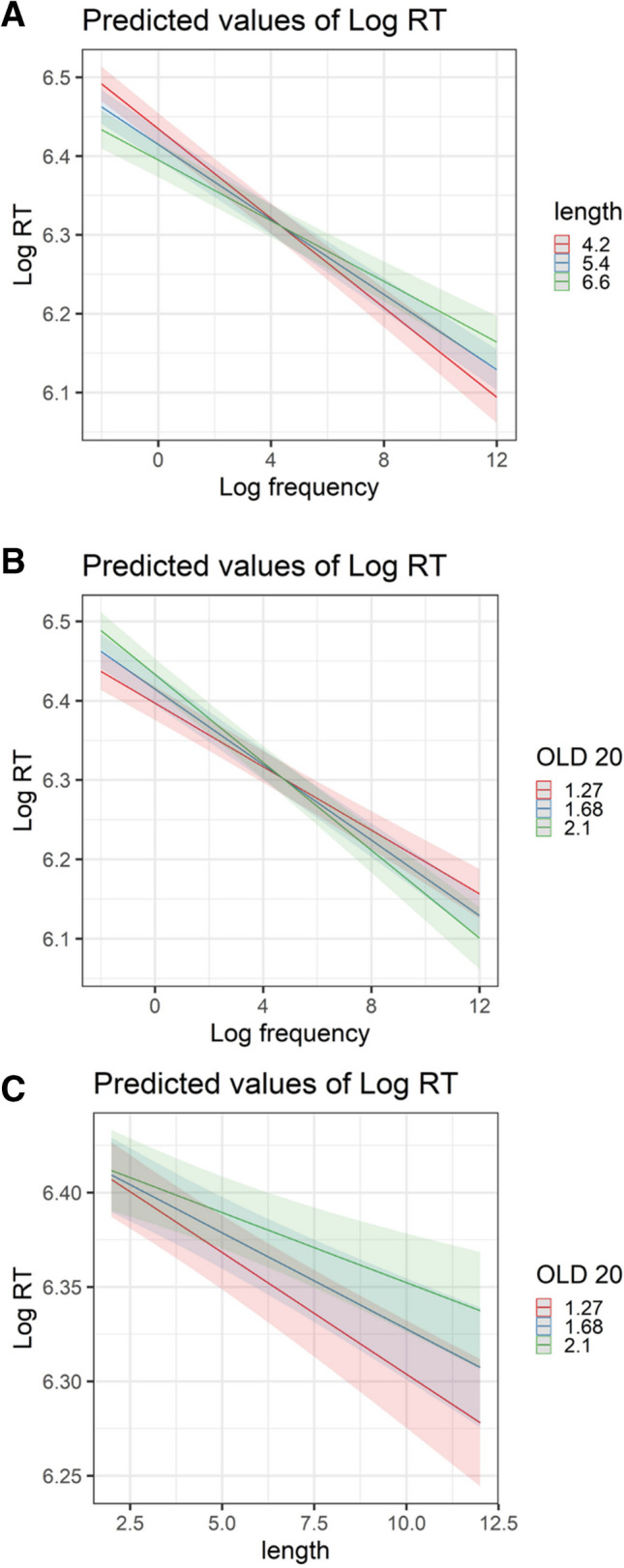
Visual depiction of significant Interactions in the *RT model, LD data.* **A** Interaction between word length and log frequency. **B** Interaction between OLD20 and log frequency. **C** Interaction between OLD20 and word length

In terms of general predictors, as expected, there was a significant effect of log frequency (*t*(470.8) =  − 23.85, *p* < 0.001), with more frequent words recognized faster. There was a significant word length effect (*t*(320.9) =  − 6.06, *p* < 0.001), where participants recognized longer words faster than shorter ones. This suggests that converging on a lexical candidate is easier for longer words in Hebrew, potentially because longer words contain vowel letters and incur less phonological ambiguity. However, we note that when OLD20 was removed from the model, the length effect was no longer significant (see Supplementary Materials S2). There was a significant OLD20 effect (*t*(750.8) = 6.35, *p* < 0.001), indicating that participants responded faster to words having many orthographic neighbors (i.e., smaller OLD20). All included interactions were significant. Inspecting the visual depictions of these interactions (Fig. 6) revealed that in low-frequency words, participants responded faster to longer than to shorter words, but this pattern was reversed in high-frequency words, where shorter words were recognized faster than longer words (Fig. 6A). As for interactions with the OLD20 effect, participants responded faster to low-frequency words with many orthographic neighbors, but slower to high-frequency words with many neighbors (Fig. 6B). Also, participants showed an OLD20 effect in the typical direction (i.e., faster responses in lower OLD20, which means more neighbors) only in longer words, whereas this effect was absent in short words (Fig. 6C).

We now turn to the central Hebrew-specific predictors. There was a main effect of Semitic structure, where participants responded faster to Semitic words (*t*(562.1) =  − 4.76, *p* < 0.001). There was also a significant effect of clitics, where responses were slower to words with one or two clitics than to words without clitics: Fixed effects reflecting the effect of a single clitic (*t*(4,072) = 4.74, *p* < 0.001) and two clitics (*t*(4,027) = 3.38, *p* < 0.001) were significant and positive, although no effect was found for three clitics (*t*(4,254) = 0.29, *p* = 0.77). This suggests that the presence of clitics requires a process of decomposition. Pronunciation entropy was also significant (*t*(413.3) = 4.17, *p* < 0.001), with participants responding more slowly to words with greater ambiguity. We return to discuss this finding below.

**Accuracy model.** The logistic mixed-effects model that converged included by-subject random slopes for word length, frequency, semitic tagging, and OLD20, in addition to by-subject and by-item random intercepts.

**Fig. 7:**
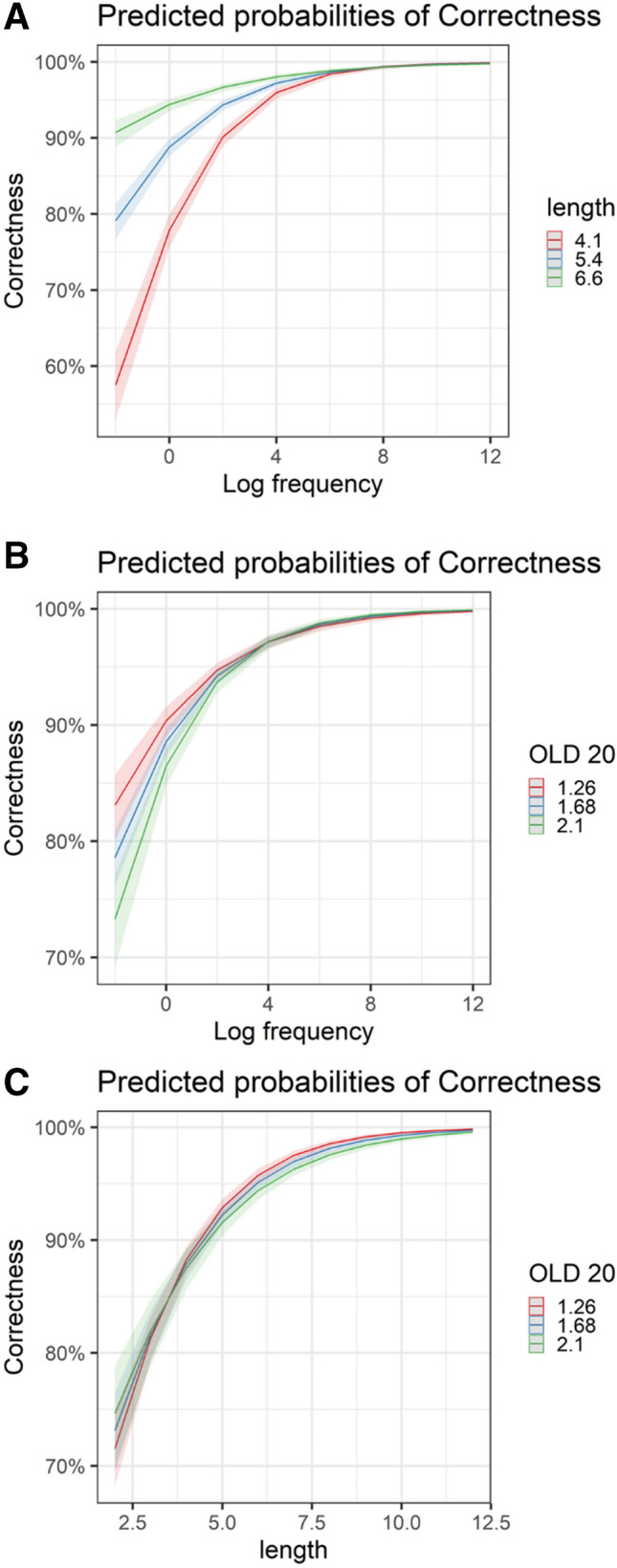
Visual depiction of significant Interactions in the *accuracy model, LD data*. **A** Interaction between word length and word log frequency. **B** Interaction between OLD20 and word log frequency.** C** Interaction between OLD20 and word length

In terms of general predictors, participants were more accurate in identifying frequent words (*Z* = 21.72, *p* < 0.001) and longer words (*Z* = 20.31, *p* < 0.001), and were more accurate when a word had many orthographic neighbors (*Z* =  − 3.51, *p* < 0.001; however, running the model without word length resulted in a “flipped” OLD20 effect, see Supplementary Materials S2). All interactions were again significant (Fig. 7). Low-frequency shorter words incurred more errors than longer words, whereas for high-frequency words, word length had no effect (see Fig. 7A). Also, among low-frequency words, having many orthographic neighbors incurred fewer errors, while for high-frequency words, OLD20 scores had no impact (Fig. 7B). Lastly, for longer words, having many orthographic neighbors incurred fewer errors (Fig. 7C).

Considering the Hebrew-specific predictors, there was a significant difference between accuracy rates in response to Semitic versus non-Semitic words, with better accuracy in responses to Semitic words (*Z* = 5.77, *p* < 0.001). More errors were found for words with a single clitic than for those with no clitics (*Z* =  − 3.59, *p* < 0.001), with no significant effects for two (*Z* =  − 0.42, *p* = 0.67) or three clitics (*Z* =  − 1.26, *p* = 0.21). Lastly, mirroring the RT data, participants had higher error rates in words with higher pronunciation entropy (*Z* =  − 2.31, *p* = 0.02).

#### Evidence from the naming task

**RT model.** The maximal random-effects structure that converged for this model included by-subject random slopes for word length, frequency, pronunciation entropy, semitic tagging, and OLD20, as well as by-subject and by-item random intercepts.

**Fig. 8:**
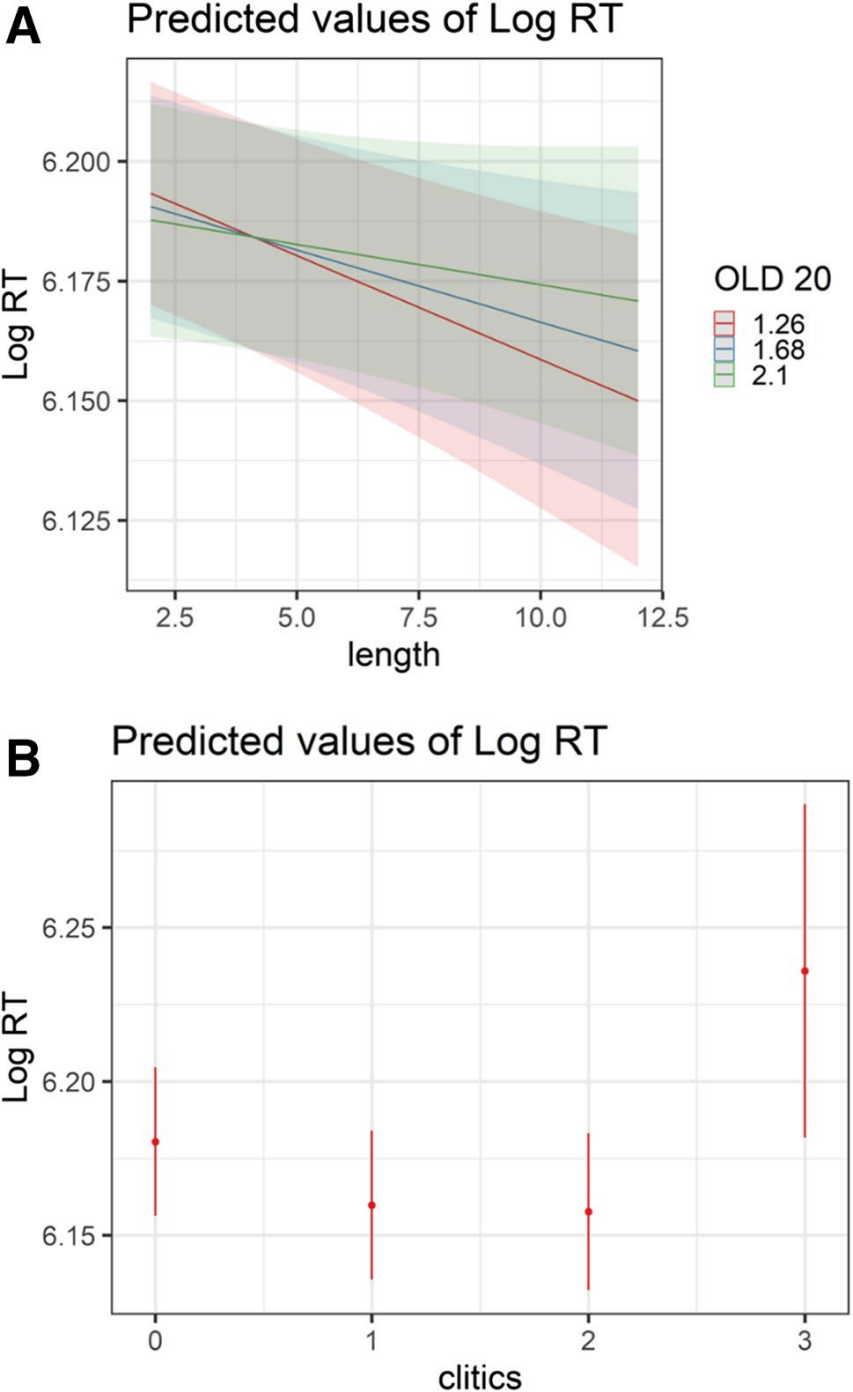
Visual depiction of effects of interest in the *RT model, naming data.* **A** Interaction between OLD20 and word length (the only significant interaction in this model). **B** Estimated mean log-transformed RT for words as a function of the number of clitics

Similar to the LD RT model, participants’ responses were faster in naming more frequent words (*t*(680.8) =  − 11.89, *p* < 0.001) and longer words (*t*(463.3) =  − 2.3, *p* = 0.02). The direction of the word length effect again suggests that longer words in Hebrew incur less uncertainty regarding word recognition. There was no significant effect of OLD20 (*t*(1024) = 1.01, *p* = 0.28). Considering interactions, only the interaction between word length and OLD20 was significant (*t*(3,990) = 3.11, *p* = 0.002), showing faster naming of words with more neighbors for longer but not for shorter words (see Fig. 8A).

We turn again to the Hebrew-specific predictors. Similar to the LD task, participants named Semitic words faster than non-Semitic words (*t*(554.7) =  − 2.17, *p* = 0.03). There were significant effects of clitics for words with a single clitic (*t*(3,899) =  − 11.95, *p* < 0.001), two clitics (*t*(3,945) =  − 5.05, *p* < 0.001), and three clitics (*t*(4,569) = 2.24, *p* = 0.02). However, in contrast to the LD model, participants responded *faster* to words with one or two clitics than to words without clitics. Since RTs in the naming task are recorded once the initial sound is uttered, this suggests that, potentially given their distributional properties, clitic letters are decomposed and pronounced initially and rapidly. However, an opposite direction was revealed for words with three clitics, which incurred slower responses (see Fig. 8B), suggesting that clitic decomposition is complex once there is a high number of clitic letters. Finally, as predicted, participants were slower to name words with greater pronunciation entropy (*t*(494.7) = 6.08, *p* < 0. 001).

**Accuracy model.** The logistic mixed-effects model that converged included by-subject random slopes for word length in addition to by-subject and by-item random intercepts.

**Fig. 9:**
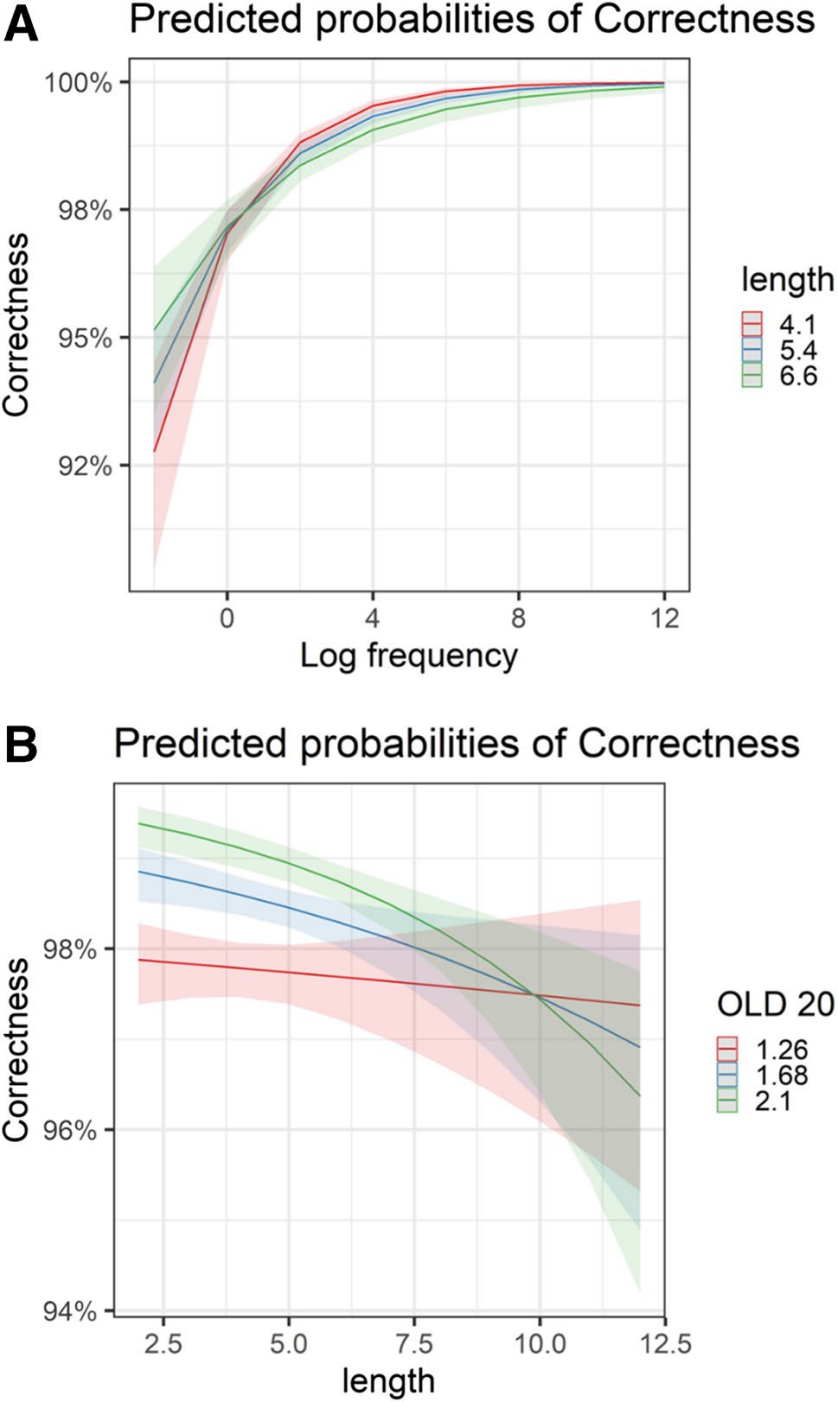
Visual depiction of significant Interactions in the *accuracy model, naming data.* **A** Interaction between word length and word log frequency. **B** Interaction between OLD20 and word length

Participants again showed the expected frequency effect (*Z* = 15.67, *p* < 0.001). However, in contrast to the LD results, they made fewer naming errors in shorter words (*Z* =  − 2.75, *p* = 0.006; although, when removing OLD20 from the model, the length effect flipped, see Supplementary Materials S2). There was a significant OLD20 effect, indicating that participants were more accurate when a word had fewer orthographic neighbors (*Z* = 7.26, *p* < 0.001). In terms of interactions (Fig. 9), there was a significant interaction between word length and OLD20 (*Z* =  − 6.32, *p* < 0.001): participants made more errors naming short words with many orthographic neighbors, and made more errors naming longer words with few orthographic neighbors. There was also a significant interaction between log frequency and word length (*Z* =  − 3.02, *p* = 0.003), with length effects revealed only for words in the mid-frequency range and above (but not in low-frequency words).

As for Hebrew-specific predictors, there was no difference in naming accuracy between Semitic and non-Semitic words (*Z* = 0.63, *p* = 0.53). Participants made more errors reading words with one (*Z* =  − 4.75, *p* < 0.001), two (*Z* =  − 2.87, *p* = 0.004), and three clitics (*Z* =  − 3.29, *p* < 0.001) than words without clitics. Also, as expected, participants made more errors reading words with higher pronunciation entropy (*Z* =  − 3.71, *p* < 0.001).

## Discussion

How the basic characteristics of writing systems impact visual word recognition behavior has been the focus of extensive research (see, e.g., Frost, 2012, for review and discussion). In this vein, studies conducted in different languages, particularly those including large-scale LPs, have provided important insights regarding the different computations readers employ during reading in their writing system, highlighting high-order principles of word recognition. From this perspective, evidence from Hebrew has continuously shaped theories and models of reading, given the unique characteristics of its writing system. However, word recognition studies in Hebrew to date have employed small, targeted experiments, covering only a limited part of the language’s lexicon. To address this gap, we present here for the first time a large-scale dataset of reading behavior in a Semitic language, Hebrew, comprising LD responses to 10,000 words and nonwords, and naming responses to 5000 words. In this first paper, we then utilize the data from the Hebrew LP (HeLP) to examine the contribution of general predictors (lexicality, frequency, length, and orthographic neighborhood), and Hebrew-specific predictors (Semitic structure, clitic letters, and extent of phonological ambiguity), to visual word recognition performance. As we discuss below in detail, our findings offer important insights regarding the computations involved in the processing of printed words in a writing system such as Hebrew, suggesting a set of universal computations involved in print processing.

### General predictors of word recognition

Unsurprisingly, the benchmark effects of frequency and lexicality emerged in Hebrew as in any LP, confirming that these principles of lexical search are similar across writing systems. Of theoretical interest, therefore, are findings in which Hebrew seems to diverge from the well-researched European languages.

A first finding of interest is the effect (or lack thereof) of word length, mainly in LD (the parallel effects in naming are somewhat weaker). When interpreting the HeLP findings for length, an important factor to consider is the collinearity between word length and OLD20 (*r* = 0.79). That shorter words have more orthographic neighbors is typical of many writing systems, as revealed in other LPs (e.g., a correlation of 0.77 in French, Ferrand et al., 2010; a correlation of 0.79 in European Portuguese, Soares et al., 2019). But note that in the vast majority of LPs, length effects were significant even when this high correlation was partialed out in the analyses. For example, in the Malay LP, the several length measures that were used showed correlations ranging from 0.47 to 0.77 with the OLD20 scores, and yet, independently, word length was the strongest predictor of performance in both LD and naming tasks (Yap et al., 2010). In light of those previous results, the consistent lack of word length effect for words in Hebrew stands out, and models that also included OLD20 often revealed a reversed length effect, where longer words incurred faster recognition time and greater accuracy. Compared with other existing LPs, these results seem to align with findings in the Persian LP (Nemati et al., 2022). A possible common factor to Hebrew and Persian is that in both languages, most vowels are omitted from orthographic representation of words. Since longer words on average include more vowel letters, they incur less phonological ambiguity, pointing to a lexical candidate more rapidly and resulting in faster recognition.^4^ More broadly, our findings resonate with previous claims that word length effects are stronger in more transparent writing systems (e.g., Cuetos & Suarez-Coalla, 2009; Ellis & Hooper, 2001; and see Weiss et al., 2015, for related evidence from pointed vs. un-pointed Hebrew). Our results go a step further to suggest that in Hebrew, word length effects are sometimes *reversed*, arguably due to lower levels of phonological ambiguity in longer words.

A second point of interest is the different impact of orthographic neighbors in the LD versus the naming task. While the typical facilitatory effect of orthographic neighborhood was observed in LD, our findings show either that orthographic neighborhood was not predictive of naming RT and accuracy, or that having many orthographic neighbors of a word resulted in an inhibitory effect. This contrasts with studies showing that the presence of many orthographic neighbors has a facilitatory effect on naming English words (e.g., Yarkoni et al., 2008). Importantly, there are documented cross-linguistic differences in orthographic neighborhood effects in naming. For example, similar to our present finding in Hebrew, Chang et al. (2016) reported an inhibitory effect of neighborhood size in Chinese, and demonstrated through computational modeling that the division of labor between phonological and semantic pathways in deep orthographies is the key to accounting for the inhibitory effect of neighborhood size (and see Peereman & Content, 1997, for differences between English and French). The difference between LD and naming, then, reflects substantial differences in computations in a deep orthography like Hebrew. While the presence of many orthographic neighbors could contribute to a fast decision of whether a letter string is a Hebrew word or not, naming requires one to identify, select, and pronounce specific lexical candidates that often differ in vowel configurations. Hence, for Hebrew, the presence of many competitors seems to slow response times, rather than accelerate them.

### Hebrew-specific effects

Given the unique properties of Hebrew, understanding the role of the Hebrew-specific predictors provides important insights with regard to visual word recognition in that language. Hence, in this section we review findings pertaining to the three Hebrew-specific predictors tested: Semitic structure, presence of clitics, and phonological ambiguity.

With regard to Semitic structure, even though the Semitic tagging we employed was conservative (defining a word as “clearly Semitic” only if its root letters were clearly productive), 75% of the 5000 words were tagged as such. Most of our statistical models showed that participants’ performance improved when presented with words having a Semitic structure. The present findings of HeLP indicate then that Hebrew readers become attuned to the statistical properties of Hebrew with its non-concatenated morphology, and are more efficient in processing Hebrew words when they conform to the highly prevalent Semitic form of intertwined root and word pattern morphemes. We assume that through statistical learning, readers become increasingly efficient in detecting the root letters within printed Semitic words, enabling the fast decomposition of printed words into their constituent morphemes (see, e.g., Feldman et al., 1995; Velan et al., 2013). This provides readers with the missing vowel information, and leads to fast lexical access when words are organized by morphological rather than simple orthographic principles (see Frost et al., 2005, for discussion).

Regarding the effects of clitics, our findings indicate that the number of clitic letters impacted performance, with responses to words with clitics generally being less accurate and incurring slower RTs. This finding suggests that when words appear in isolation without disambiguating context, clitic letters add further complexity to the process of decomposing the printed word into its morphemic constituents. An interesting deviation from this pattern, however, was found in the naming task: participants read aloud words with clitics *faster* (although still with more errors). Given the high prevalence of clitics and the relative systematicity of their pronunciation (e.g., “and” in most cases is pronounced as \ve\, “the” as \ha\, etc.), and in light of the time constraints in the naming task, we cannot rule out the possibility that participants initiated the pronunciation of the initial clitics before fully recognizing the word (and the higher error rate for words with clitics indeed supports this explanation). As naming latencies reflect the time course of the initial utterance, our overall speeded responses in the naming task could reflect participants’ high confidence in initiating a vocal response to the initial clitic letter.

The third important characteristic of Hebrew is its phonological under-specification. While previous research simply counted the number of empty vowel slots to assess phonological uncertainty (e.g., Frost, 1995), in the present work we captured the level of phonological uncertainty when pronouncing a word by mathematically quantifying phonological entropy across responses in the full sample of participants (see also De Simone et al., 2021). Our results show that in all models, higher pronunciation entropy hindered performance, for both naming and LD. Whereas the impact of phonological entropy in naming is anything but surprising, the parallel finding for LD is striking. The orthographical depth hypothesis (Frost et al., 1987) has argued that readers of shallow orthographies rely strongly on phonological cues in visual word recognition, while readers of deep orthographies such as English or Hebrew rely more on orthographic or semantic cues. It was assumed that in deep orthographies, the phonological information of a word is mediated by the internal lexicon (Frost et al., 1987; Katz & Feldman, 1981). Our present data suggests that phonological information is computed not only when pronouncing a word, but also when simply identifying it in the LD task. This finding accords with the claim that early and fast phonological computations in visual word recognition characterize the reading process in any orthography whether shallow or deep (for discussion see Frost, 1998; Rastle & Brysbaert, 2006). Indeed, Rueckl et al. (2015) have shown that readers of different languages with different orthographic depths demonstrate similar neuronal processing of printed words, including in brain areas associated with phonological processing. Our results support this line of research, showing early phonological processing in Hebrew, which is considered a highly deep orthography. That said, we should caution that our measure of phonological entropy also potentially reflects uncertainty in the mapping between print and *meaning*; this is because, in Hebrew, multiple pronunciations of the same word form also often have multiple distinct meanings, and therefore more homographic word forms also typically carry more ambiguity in the orthographic–semantic mapping. Future work should carefully disentangle the effects of different types of ambiguity by providing and validating word-level measures of print–speech and print–meaning regularities in Hebrew. We expect the HeLP data to be crucial in the validation of such measures (for parallel work in English using the ELP data, see, e.g., Chee et al., 2020; Marelli & Amenta, 2018; Siegelman et al., 2020, 2022).

### What stands out? What is universal?

As outlined in our Introduction, our theoretical approach to LPs is that they go far beyond the descriptive statistics of yet another writing system. Divergent findings in LPs point to higher-order computational principles that explicate the difference in results in one language relative to another. Here we argue that to account for the range of findings revealed in HeLP, visual word recognition should be considered as a process of uncertainty reduction with respect to the identity of lexical candidates and their phonological structure, as represented by the printed forms. This universal principle accounts for cross-linguistic differences by weighting the set of constraints that drive uncertainty reduction in a given writing system.

The main problem in reading Hebrew is in converging on an unequivocal lexical and phonological solution to a range of parsing and decoding alternatives. While reading in context significantly reduces uncertainty, leading in most cases to a single solution, words in isolation incur significant uncertainty. Uncertainty in Semitic languages concerns competing parsing possibilities (e.g., whether the initial letter is a clitic letter, a word pattern letter, or a root letter, which is critical for identifying the correct lemma) and also competing phonological representations. This perspective accounts, for example, for the faster responses to longer words, since often they incur lower entropy than shorter words. It offers a possible explanation for the faster responses to words with Semitic structure, since these words typically contain several cues for correct morphological decomposition (and see Bar-On et al., 2017, 2019, 2021, for discussions of uncertainty reduction in Hebrew).

Considering visual word recognition as a process of uncertainty reduction also outlines the range of dimensions to consider when comparing performance across languages, and generates predictions regarding cross-linguistic differences in reading. It shifts the scope of analysis from unidimensional factors such as print–speech transparency, or morphological complexity, to regard performance in visual word recognition in terms of constraint satisfaction, where multidimensional constraints interact to determine the outcome of processing.

### Future directions

Our present findings offer compelling evidence of how the unique morphological and phonological properties of Hebrew play an important role during visual word recognition. However, our present analyses are but a first step which involves coarse-grained quantification of words’ properties. Following in the steps of previous LPs, the HeLP project adheres to the principles of open science, making all data available for secondary analyses, which we hope will facilitate future investigation into the exact predictors of visual word recognition in Hebrew.

As mentioned briefly above, one important avenue for future research is the development of more subtle and precise quantification of orthographic–phonological regularities in Hebrew. In the current work, we only used a measure of pronunciation entropy, which was calculated given the actual pronunciations that participants uttered in the naming task. Although this measure has ecological validity as it includes all pronunciations that were expressed in practice by our sample of participants, it is limited in two important ways. First, there are other phonological expressions for the words we employed that were not included in the measure’s calculation. For example, the printed word "כמורה" (KMWRH) has five different phonological forms that bear meaning in Hebrew (/kmura/, /kemore/, /kemora/, /kamore/, /kamora/), but only three of them were produced by our participants. Perhaps more importantly, considering only actual pronunciations does not capture the full extent to which different graphemes predict phonemes in the language. We leave it for future work to develop precise corpus-based metrics of the links between orthography and phonology in Hebrew. This work will most likely involve adapting measures developed in English and other European languages (e.g., Chee et al., 2020; Siegelman et al., 2020) to capture the unique properties of Hebrew (e.g., the fact that in Hebrew, in contrast to English, substantial irregularities also exist in the mapping of consonant letters into phonemes, rather than mostly vowel letters).

Note that producing precise measures of orthographic–phonological entropy is but one step in assessing uncertainty in an Abjad writing system such as Hebrew. Other avenues would include the impact of phonological Levenshtein distance, as well as measures of orthographic–semantic regularities (e.g., Marelli & Amenta, 2018; Siegelman et al., 2022). Multiple other avenues of analyses using the HeLP data can replicate studies using the ELP data in a writing system with a divergent structure, including those in psycholinguistic ratings such as word concreteness, (Brysbaert et al., 2014), age of acquisition (Kuperman et al., 2012), and body–object interaction (Pexman et al., 2019), to name a few. Future analyses should also consider the possible interactions that may exist between Hebrew-specific predictors and general psycholinguistic properties (e.g., the fact that Semitic structure or extent of phonological ambiguity may impact processing differently across word lengths). Merging all these dimensions together would enable the alignment of writing systems for cross-linguistic comparisons, = while simultaneously considering the possible interactions of their orthographic, phonological, and semantic properties.

## Supplementary Information

Below is the link to the electronic supplementary material.

Supplementary file1 (DOCX 262 KB)

## Data Availability

The full HeLP data are available via the Open Science Framework (OSF) website for secondary data analyses, along with the full lists of items used in the studies: https://osf.io/nxq8g/.

## Footnotes

1 Hebrew also has a diacritical variant of the writing system, where vowels are depicted by points (appearing mostly under printed letters). These vowel marks are typically taught in the first grade, assisting teachers in developing decoding skills during reading acquisition, but starting from the end of the second grade, printed and written Hebrew does not normally include diacritical marks (see Share & Bar-On, 2018, for a detailed discussion). Apart from poetry and religious texts, adult reading material is always un-pointed.

2 Sixteen nonwords were accidently used twice, each of them appearing in two separate sublists of the LD task. Hence, these 16 nonwords had data on twice as many participants as other stimuli. The vast majority of items, however, including all word stimuli, had between 29 and 40 observations per item: only 0.2% of datapoints (1204 out of 747,595 trials in the full dataset) were from the 16 repeated nonwords.

3 Session number was included as a covariate to account for the repeated testing of some participants across multiple sessions. However, one may still wonder whether repeated testing interacts with key psycholinguistic variables, potentially biasing the results reported below. To alleviate this concern, in Supplementary Materials S3 we reran the central models on data from the first session of each participant only. Showcasing the robustness of the reported effects, the findings were highly similar (in terms of both the estimated coefficients and their significance) to the data from all sessions reported in the main text. See Supplementary Materials S3 for details.

4 Some readers may wonder why the correlation between word length and phonological entropy, while negative, is quite small, *r* =  − 0.16 (Table 3 above). In this context, it is important to keep in mind that our measure of phonological entropy reflects the number of meaningful pronunciations a word has, given the heterophonic aspect of Hebrew. As such, it does not fully capture a word’s degree of phonological ambiguity (i.e., uncertainty or inconsistency): It does not reflect the difficulty in generating unique pronunciations given the number of missing vowels in the orthographic sequence. Hence two words can have a unique lexical pronunciation (and hence a similar score in the measure of phonological entropy), but one with few missing vowels and one with many.

## Code availability

The project’s OSF repository also includes the code used for analyses reported in this paper.
