# Supplementary material for: HeLP: The Hebrew Lexicon project
Source: Behav Res Methods. 2024 Sep 9;56(8):8761–83. doi: 10.3758/s13428-024-02502-4 (PMC11525262; doi:10.3758/s13428-024-02502-4)
Supplement: Supplementary file 1 — Supplementary file1 (DOCX 262 KB) [file 13428_2024_2502_MOESM1_ESM.docx]

**Supplementary Materials for: Stein et al., “HeLP: The Hebrew Lexicon Project”**

**Supplementary Materials S1: Basic Effects: Lexicality, Word Length, and Neighborhood Density**

***RT model- All 20,000 stimuli***

Basic effects were examined on all 10,000 words and 10,000 nonwords using a linear mixed effects model. Log RT served as the dependent variable, with fixed-effects of lexicality (word/nonword), word-length, OLD20 and the number of session this was for each participant. The two-way and three-way interactions between lexicality, word-length and OLD20 were also included. Continuous predictors were scaled, and lexicality was effect-coded. The maximal model that converged included by-subject and by-item random intercepts.

The fixed-effect estimates are summarized in Table S1. A significant Lexicality effect was found (t(18,860)=-28.73, *P*<0.001), indicating faster RT for words than nonwords. Length effect was found as well (t(720.9)=19.18, *P*<0.001), indicating overall faster reactions in response to shorter stimuli (although the length effect was modulated by lexicality. See below). There was an OLD20 effect (t(18,590)=-15.12, *P*<0.001), which suggests that participants' RTs are faster when there are few orthographic neighbors to a stimulus. Also, as expected, participants responded faster with every additional session they took part in (t(77690)=-52.53, *P*<0.001).

In addition to the main effects, all interactions were significant. In particular, there were important differences between words and nonwords in the effect of length and orthographic neighborhood size (Figure S1). Thus, participants responded faster to shorter nonwords, especially if they had little orthographic neighbors. In contrast, participants responded faster to longer words, especially if they had many orthographic neighbors.

|  | **RT** | | | **Accuracy** | | |
| --- | --- | --- | --- | --- | --- | --- |
| **Predictors** | **Coefficient** | **T value** | **P value** | **Coefficient** | **Z value** | **P value** |
| **Length** | 0.026 | 19.180 | < 0.001 *** | 0.121 | 7.786 | < 0.001 *** |
| **OLD20** | -0.017 | -15.120 | < 0.001 *** | 0.119 | 6.562 | < 0.001 *** |
| **Lexicality** | -0.022 | -28.730 | < 0.001 *** | -0.131 | -4.273 | < 0.001 *** |
| **Lexicality x length** | -0.038 | -41.970 | < 0.001 *** | 0.532 | 38.809 | < 0.001 *** |
| **Lexicality x OLD20** | 0.063 | 56.220 | < 0.001 *** | -0.812 | -48.414 | < 0.001 *** |
| **Length x OLD20** | 0.004 | 11.290 | < 0.001 *** | -0.043 | -7.976 | < 0.001 *** |
| **Length x OLD20 x Lexicality** | -0.005 | -14.930 | < 0.001 *** | 0.066 | 12.322 | < 0.001 *** |
| **Session number** | -0.029 | -52.430 | < 0.001 *** | -0.115 | -14.432 | < 0.001 *** |

**Table S1.** LD linear and logistic mixed effect models results. calculated on 10,000 words and 10,000 nonwords.


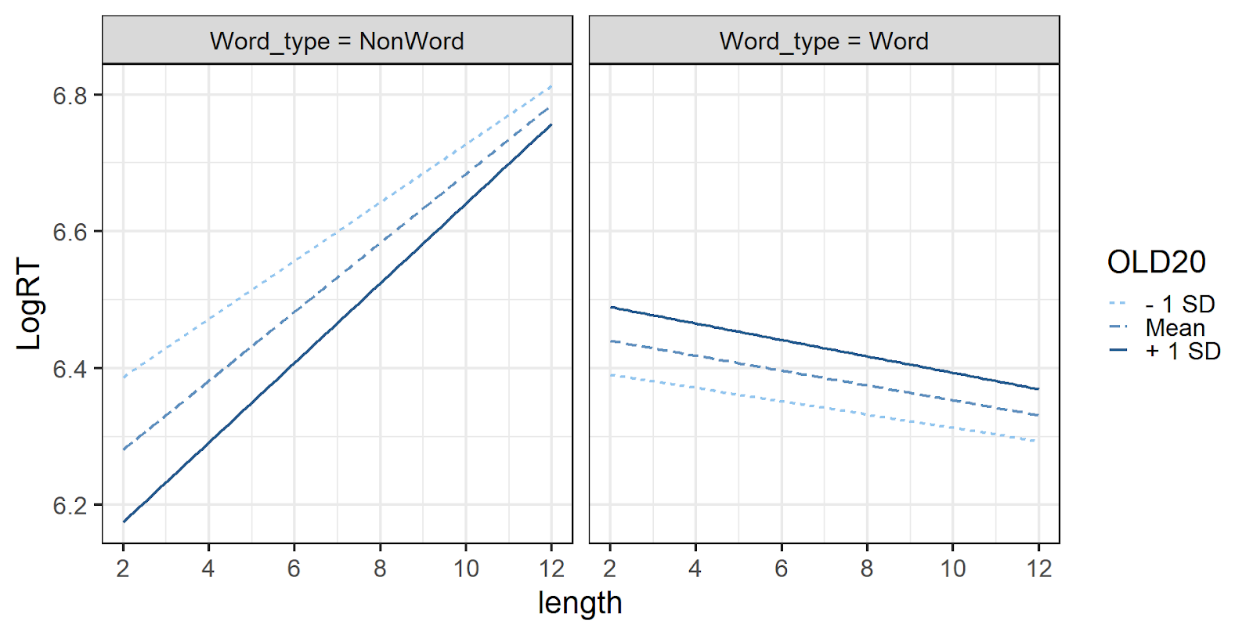


**Figure S1.** The estimated three-way interaction between lexicality (words vs. nonword), length, and OLD20 in predicting LD log-transformed RT.

***Accuracy model- All 20,000 stimuli***

The basic effects were examined on all 10,000 words and 10,000 nonwords using a logistic mixed effects model. Accuracy was predicted using the same fixed effects as the linear model above, with the maximal model that converged again including by-participant and by-item random intercepts. The fixed-effect estimates are shown in Table S1. As in the linear RT model, Lexicality had a significant effect (Z=-4.27, *P*<0.001), reflecting lower accuracy for words than nonwords. A significant word-length effect was found (Z=7.78, *P*<0.001), indicating *less* errors for longer stimuli (see more in the main text on this topic). The effect of OLD20 (Z=6.56, *P*<0.001) suggests that participants made less errors when stimuli had few orthographic neighbors compared to stimuli that had many orthographic neighbors. Session number was significant as well (Z=-14.43, *P*<0.001), with accuracy decreasing with the number of sessions. All interactions were again significant, mirroring the effects in the RT model (See Figure S2). In particular, participants made more errors identifying nonwords the longer they were, and the more orthographic neighbors they had. As for words, more errors were made in shorter words, and especially words with fewer orthographic neighbors.


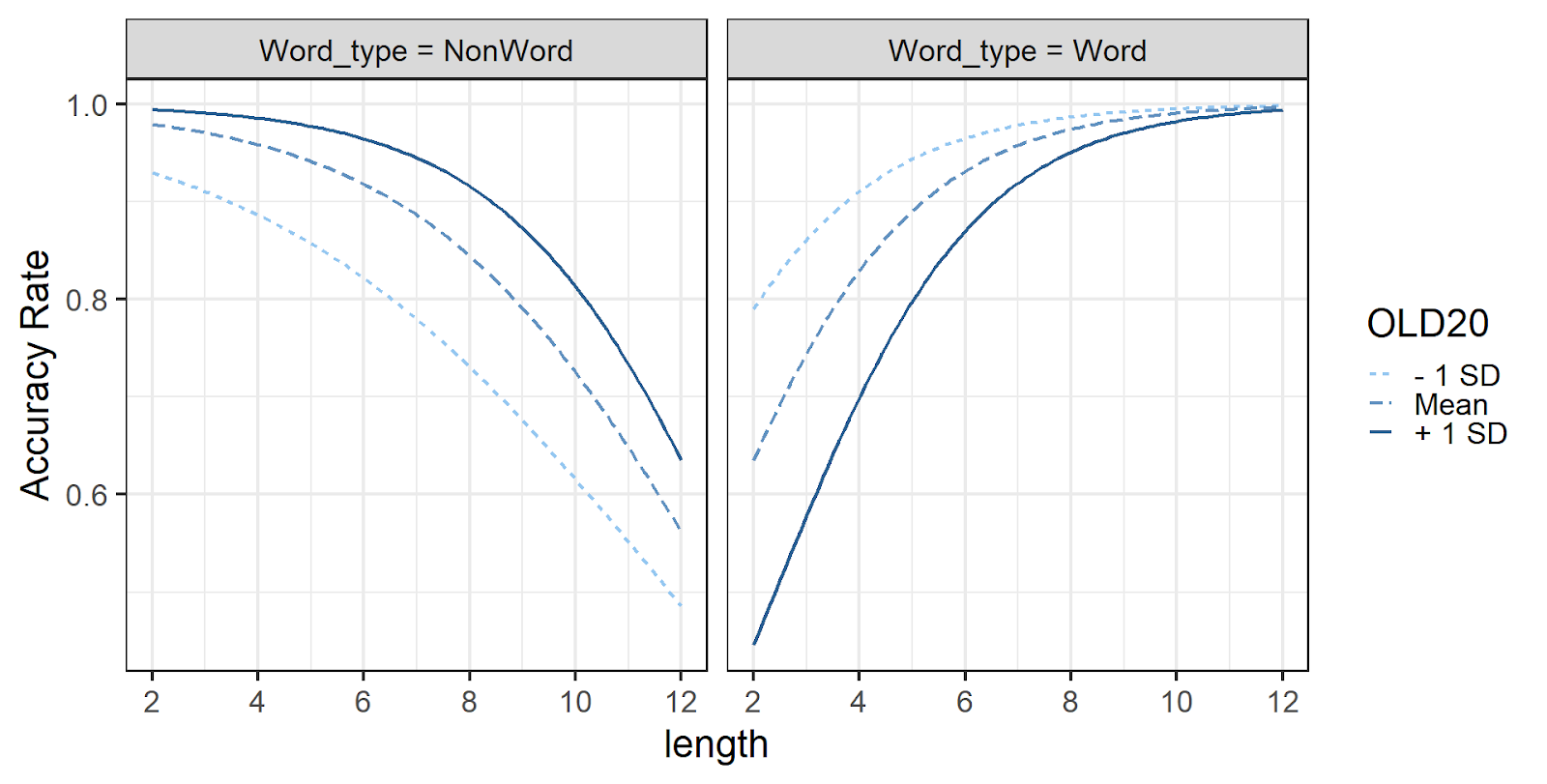


**Figure S2.** The estimated three-way interaction between lexicality (words vs. nonword), length, and OLD20 in predicting LD accuracy.

**Supplementary Materials S2: Isolating the Effects of OLD20 and Word Length**

Tables S2 and S3 report the results of a series of models conducted on the dataset of the 4,441 words in the LD and naming tasks. The goal of these models was to examine whether the effects of word length and orthographic neighborhood (i.e., OLD20) are observed even when they are inserted into the models separately, to confirm that they are not solely an outcome of the high collinearity between these two predictors. Other than the exclusion of one of the fixed effects each time (i.e., running each model without either length or OLD20), the specification of all models remained identical to those in the main text.

In the RT models word naming models, there were no qualitative changes in the results of the models without OLD20 or length. In predicting accuracy in the naming data, excluding length from the model led to no qualitative changes in effects; however, excluding OLD20 led to a “flipping” in the direction of the length effect on accuracy, which was now estimated to be positive (i.e., longer words have higher accuracy), rather than negative, as observed when orthographic neighborhood is controlled for.

In the LD models, for RT, the exclusion of OLD20 resulted in a now-insignificant effect of length. The exclusion of length resulted in similar effects as the original models. For LD accuracy, the exclusion of length led to a “flipping” in OLD20, now estimated to be positive (i.e., higher OLD20, higher accuracy) rather than negative. The exclusion of OLD20 did not qualitatively impact the length effect.

Taken together, the results of these additional models suggest that the effects of length and OLD20 reported in the main paper are generally stable, and in most cases are not the outcome of high collinearity alone. At the very least, these models suggest that the effects of length generally diverge in Hebrew from the “typical” pattern observed in many European languages, where longer words are associated with slower and less accurate responses. However, in some cases, targeted experiments are clearly needed to better isolate the effects of these two predictors. See further discussion in the main text.

**Table S2.** Models re-estimating the effects on LD and naming RT and accuracy with the exclusion of the word length predictor. **p* < .05; ***p <* .01; ****p* < .001

|  | **Lexical Decision: RT** | | | **Lexical Decision: Accuracy** | | | **Naming: RT** | | | **Naming: Accuracy** | | |
| --- | --- | --- | --- | --- | --- | --- | --- | --- | --- | --- | --- | --- |
|  | **Coefficient** | **T value** | ***p*-value** | **Coefficient** | **Z value** | ***p*-value** | **Coefficient** | **T value** | ***p*-value** | **Coefficient** | **Z value** | ***p*-value** |
| **Log Frequency** | -0.035 | -29.283 | < 0.001 *** | 0.575 | 22.425 | < 0.001 *** | -0.014 | -12.097 | < 0.001 *** | 0.502 | 15.465 | < 0.001 *** |
| **OLD20** | 0.005 | 2.925 | 0.004 ** | 0.306 | 12.560 | < 0.001 *** | 0.000 | 0.152 | 0.879 | 0.102 | 3.477 | < 0.001 *** |
| **semitic** | -0.016 | -6.537 | < 0.001 *** | 0.392 | 9.242 | < 0.001 *** | -0.007 | -2.960 | 0.003 ** | 0.074 | 1.227 | 0.220 |
| **clitic-1** | 0.004 | 2.188 | 0.029 * | 0.125 | 3.891 | < 0.001 *** | -0.023 | -13.642 | < 0.001 *** | -0.303 | -5.993 | < 0.001 *** |
| **clitic-2** | 0.011 | 2.045 | 0.041 * | 0.241 | 2.750 | 0.006 ** | -0.025 | -5.663 | < 0.001 *** | -0.407 | -3.096 | 0.002 ** |
| **clitic-3** | 0.003 | 0.116 | 0.908 | -0.254 | -0.589 | 0.556 | 0.055 | 2.201 | 0.028 * | -1.985 | -3.317 | < 0.001 *** |
| **Pronunciation entropy** | 0.005 | 5.135 | < 0.001 *** | -0.070 | -4.617 | < 0.001 *** | 0.006 | 6.532 | < 0.001 *** | -0.100 | -4.197 | < 0.001 *** |
| **Log Frequency x OLD20** | -0.003 | -3.141 | 0.002 ** | 0.027 | 1.467 | 0.142 | -0.001 | -1.337 | 0.181 | 0.022 | 0.763 | 0.446 |
| **session number** | -0.029 | -27.021 | < 0.001 *** | -0.168 | -11.065 | < 0.001 *** | -0.006 | -7.741 | < 0.001 *** | 0.132 | 6.823 | < 0.001 *** |

**Table S3.** Models re-estimating the effects on LD and naming RT and accuracy with the exclusion of the OLD20 predictor. **p* < .05; ***p <* .01; ****p* < .001

|  | **Lexical Decision: RT** | | | **Lexical Decision: Accuracy** | | | **Naming: RT** | | | **Naming: Accuracy** | | |
| --- | --- | --- | --- | --- | --- | --- | --- | --- | --- | --- | --- | --- |
|  | **Coefficient** | **T value** | ***p*-value** | **Coefficient** | **Z value** | ***p*-value** | **Coefficient** | **T value** | ***p*-value** | **Coefficient** | **Z value** | ***p*-value** |
| **Log Frequency** | -0.037 | -26.982 | < 0.001 *** | 0.559 | 24.011 | < 0.001 *** | -0.014 | -13.438 | < 0.001 *** | 0.447 | 14.726 | < 0.001 *** |
| **Word length** | -0.001 | -0.624 | 0.533 | 0.473 | 20.718 | < 0.001 *** | -0.001 | -1.178 | 0.240 | 0.071 | 2.205 | 0.027 * |
| **semitic** | -0.019 | -7.797 | < 0.001 *** | 0.340 | 8.397 | < 0.001 *** | -0.007 | -3.155 | 0.002 ** | 0.042 | 0.676 | 0.499 |
| **clitic-1** | 0.005 | 2.318 | 0.021 * | -0.037 | -1.178 | 0.239 | -0.022 | -12.957 | < 0.001 *** | -0.297 | -5.446 | < 0.001 *** |
| **clitic-2** | 0.014 | 2.629 | 0.009** | 0.032 | 0.378 | 0.705 | -0.024 | -5.317 | < 0.001 *** | -0.388 | -2.805 | 0.005 ** |
| **clitic-3** | 0.020 | 0.706 | 0.480 | -0.692 | -1.674 | 0.094 . | 0.058 | 2.361 | 0.018 * | -1.981 | -3.207 | 0.001 ** |
| **Pronunciation entropy** | 0.004 | 4.283 | < 0.001 *** | -0.038 | -2.647 | 0.008 ** | 0.006 | 6.276 | < 0.001 *** | -0.117 | -4.676 | < 0.001 *** |
| **Log Frequency**  **x word length** | -0.001 | -0.658 | 0.510 | -0.035 | -2.206 | 0.027* | 0.000 | -0.518 | 0.605 | -0.042 | -1.473 | 0.141 |
| **session number** | -0.029 | -26.756 | < 0.001 *** | -0.172 | -11.547 | < 0.001 *** | -0.006 | -7.798 | < 0.001 *** | 0.142 | 6.918 | < 0.001 *** |

**Supplementary Materials S3: Estimating the Effects of Psycholinguistic Predictors in the First Session Only.** Table S4 below summarizes additional models that examine the impact of the various psycholinguistic predictors in the first session only, and compare them to the effects found across all sessions (reported in the paper). These analyses reveal very high agreement between the two sets of analyses: Both in terms of the estimated coefficients, as well as in their significance (i.e., out of 36 significant effects on the full data, only one failed to reach significance in the session 1 data; and all effects that were significant in the session 1 data were also significant in the full data). This finding alleviates potential concerns regarding unknown interactions between repeated testing and the tested psycholinguistic variables.

**Table S4.** Comparison of coefficients and significance levels for effects in all sessions and in the first session only. **p* < .05; ***p <* .01; ****p* < .001

|  | **Lexical Decision: RT** | | **Lexical Decision: Accuracy** | | **Naming: RT** | | **Naming: Accuracy** | |
| --- | --- | --- | --- | --- | --- | --- | --- | --- |
|  | **Coefficient – all sessions** | **Coefficient – session 1 only** | **Coefficient – all sessions** | **Coefficient – session 1 only** | **Coefficient – all sessions** | **Coefficient – session 1 only** | **Coefficient – all sessions** | **Coefficient – session 1 only** |
| **Log Frequency** | -0.035*** | -0.036*** | 0.532*** | 0.493*** | -0.014*** | -0.013*** | 0.541*** | 0.561*** |
| **word length** | -0.012*** | -0.011*** | 0.596*** | 0.613*** | -0.004* | -0.004** | -0.126** | -0.167*** |
| **OLD20** | 0.012*** | 0.008*** | -0.108*** | -0.122** | 0.002 | 0.002 | 0.361*** | 0.397*** |
| **semitic** | -0.012*** | -0.012*** | 0.235*** | 0.228*** | -0.005* | -0.006** | 0.040 | 0.068 |
| **clitic-1** | 0.010*** | 0.015*** | -0.114* | -0.148** | -0.021*** | -0.019*** | -0.262*** | -0.314*** |
| **clitic-2** | 0.018*** | 0.026*** | -0.035 | -0.184 | -0.023*** | -0.024*** | -0.394** | -0.450*** |
| **clitic-3** | 0.008 | 0.046 | -0.521 | -0.571 | 0.055* | 0.052* | -1.985*** | -2.295*** |
| **Pronunciation entropy** | 0.004*** | 0.006*** | -0.033* | -0.054** | 0.006*** | 0.006*** | -0.092*** | -0.074** |
| **word length x OLD20** | 0.003*** | 0.004*** | -0.070*** | -0.078*** | 0.002** | 0.001** | -0.099*** | -0.113*** |
| **Log Frequency x word length** | 0.007*** | 0.009*** | -0.156*** | -0.129*** | 0.002 | 0.002 | -0.134** | -0.141** |
| **Log Frequency x OLD20** | -0.006*** | -0.007** | 0.076*** | 0.011 | -0.001 | -0.001 | 0.054 | 0.039 |
| **session number** | -0.029*** | N/A | -0.170*** | N/A | -0.006*** | N/A | 0.146*** | N/A |
